# Engineering filamentous potato virus X as a platform nanotechnology for nucleic acid gene delivery

**DOI:** 10.1038/s41598-025-27488-7

**Published:** 2025-12-12

**Authors:** Bryan Duoto, Michael Tong, Krister J. Barkovich, Juliane Schuphan, Prashant Mali, Nicole F. Steinmetz

**Affiliations:** 1 Aiiso Yufeng Li Family Department of Chemical and Nano Engineering, University of California San Diego, 9500 Gilman Dr., La Jolla, San Diego, CA 92093 USA; 2 Shu Chien-Gene Lay Department of Bioengineering, University of California, La Jolla, San Diego, CA 92093 USA; 3 Center for Nano-ImmunoEngineering, University of California, San Diego, La Jolla, San Diego, CA 92093 USA; 4 Moores Cancer Center, University of California, San Diego, La Jolla, San Diego, CA 92093 USA; 5 Shu and K. C. Chien and Peter Farrell Collaboratory, University of California, La Jolla, San Diego, CA 92093 USA; 6 Department of Radiology, University of California, San Diego, La Jolla, San Diego, CA 92093 USA; 7 Institut für Molekulare Biotechnologie, RWTH Aachen University, Worringer Weg 1, 52074 Aachen, Germany; 8 Department of Bioengineering, University of California, San Diego, La Jolla, San Diego, CA 92093 USA; 9 Institute for Materials Discovery and Design, University of California, San Diego, La Jolla, San Diego, CA 92093 USA; 10 Center for Engineering in Cancer, Institute for Engineering in Medicine, University of California, San Diego, La Jolla, San Diego, CA 92093 USA

**Keywords:** Biochemistry, Biological techniques, Biotechnology, Drug discovery, Nanoscience and technology

## Abstract

Nucleic acid therapeutics and gene delivery platforms have the potential to change the way we treat disease, enabling precision medicine. Advances in biotechnology have allowed for production of virtually any functional protein or peptide in the human body through the introduction of various classes of RNA, including regulatory and coding RNAs (e.g., mRNA) in the form of linear or circular RNA (circRNA). To be effective, nucleic acid therapeutics require safe, stable, and efficient delivery vehicles that protect the otherwise labile cargo from degradation, promote cellular uptake, and facilitate payload release. Lipid nanoparticles (LNPs) have enjoyed recent clinical success for the delivery of mRNA to combat disease – especially against coronavirus 2019 (SARS CoV-2) and, more recently, against high-grade ductal breast carcinoma (clinical trial) – marking significant milestones for RNA nanomedicines. As an alternative to LNPs and mammalian viral vectors, we report the use of nucleoprotein components from a filamentous plant virus, potato virus X (PVX) as an RNA platform delivery technology. Designer nucleoprotein complexes of filamentous and halo-shaped nanoparticles were obtained through self-assembled templating on custom mRNAs and circRNAs. The flexuous nature of the PVX platform enabled the packing of circRNA yielding a unique nanoparticle platform. We explored assembly from coat protein (CP) obtained from PVX particles produced in planta but also extended the design space by producing recombinant CP by bacterial expression. Controlled mixed assemblies were achieved allowing the programming of mosaic particles with integrated functionality; length control was also demonstrated, resulting in bespoke nucleic acid delivery vehicles for tunable pharmacology. Using reporter genes, we demonstrate successful transfection of eukaryotic cells and target protein expression.

**Supplementary Information:**

The online version contains supplementary material available at 10.1038/s41598-025-27488-7.

## Introduction

The convergence of mRNA therapeutics with nanotechnology represents a transformative leap in next-generation therapeutics and offers profound implications for the treatment of diverse diseases. The rapid development and global deployment of mRNA vaccines in response to the COVID-19 pandemic, as well as recent clinical successes in immunotherapy and oncology, highlight the opportunity of nucleic acid therapeutics and their delivery technologies^1^. The modularity and flexible nature of RNA make it an ideal therapeutic agent for acute infectious diseases as well as chronic diseases like cancer, neurodegeneration, cardiovascular diseases, and inflammation^2^. However, the success of RNA therapies is contingent on delivery approaches that overcome the inherent instability of RNA and enhance cellular uptake and trafficking in target cells^3^.

The stabilizing features of mRNA, such as the 5’ 7-methyl guanosine (m7G) cap and the 3’ polyadenylated (poly(A)) tail, are fundamental in preserving the molecule from premature degradation. Similarly, circRNAs originate from pre-mRNA precursors that are spliced to form covalently closed-loop structures from the joining of the 5’ and 3’ ends of the RNA molecule – a feature that makes them less susceptible to exonucleases and increases their half-lives to up to seven times compared to their linear counterparts^4^. Yet, these features alone are insufficient for ensuring the stability and efficacy of RNA therapeutics within the biological milieu. Given that RNA is a negatively charged molecule, there is need to formulate RNA into carriers to screen the negative charge and enable uptake into cells (the mammalian cell membrane is also negative charged). Synthetic and biologic nanoparticles offer an attractive platform because these can also be tailored with targeting ligands to achieve tissue-specificity. Advancements in protein design and protein-nanoparticle technologies have provided novel solutions for the encapsulation, protection, and targeted delivery of RNA molecules^5^. Through integration of RNA-protein binding sites, directed assembly into nucleoprotein complexes can be achieved. This approach mimics and leverages the evolutionary adaptations of viruses to protect and deliver their genetic material, and several proof-of-concept studies have successfully evaluated the potential of plant viruses, and their constituent virus-like particles (VLPs), to encapsulate foreign RNAs and deliver them to mammalian cells^6,7^.

Non-viral nanotechnologies and viral vectors have been deployed as nucleic acid delivery vectors. For example, liposomes and lipid nanoparticles (LNPs) have entered clinical applications for RNA interference (RNAi) therapy (Onpattro^®^) and as COVID19 vaccines^8^. Nevertheless, there is a need for development of nanocarriers with improved stability profiles that do not require the cold-chain for distribution and storage^9,10^, and nanocarriers with build-in cell-specific targeting to realize RNA as therapeutic^11^. LNPs mainly accumulate in the liver due to the abundance of low-density lipoprotein receptors and thus have limited tissue- and cell-specificity – in addition to biophysical and cellular barriers that inhibit cytosolic delivery of RNA cargos^12,13^.

Mammalian viruses have made headways in genetic medicine – yet only a few vectors have been developed with most efforts focused on Adenoviruses, Retroviruses (e.g. Lentivirus), Adeno-associated viruses, and Herpes simplex virus^14^. Since viruses are the most abundant biological entities on the planet with an estimated 10^31^ viruses on Earth, this biodiversity offers tools for biotechnology and here we explore potato virus X (PVX) as a novel platform technology for nucleic acid delivery. The potential of plant viruses for gene delivery targeting eukaryotic cells has been demonstrated with a few isolated examples focused on early-stage development of tobacco mosaic virus (TMV) and cowpea chlorotic mottle virus (CCMV) for gene delivery^15,16^. PVX offers advantages given its shape and materials composition differentiates it from contemporary viral and non-viral systems: PVX virions are described as flexible, thread-like bodies measuring approximately 500–515 nanometers in length and 13 nanometers in diameter^17^. Each viral particle comprises around 1300 helically folded identical coat protein (CP) subunits enclosing a 6.4 kb genomic RNA (gRNA), with each turn of the primary helix consisting of 8.9 CP subunits. Thus PVX offersideal traits to carry large nucleic acid payloads^17^. The CPs of PVX are amenable to genetic engineering and chemical conjugation opening up new avenues for the development of customized nanocarriers tailored to specific therapeutic applications and payloads^18^.

PVX is a plant virus belonging to the *Potexvirus* genus of the family *Flexiviridae*. Its gRNA shares features with mammalian mRNA, including a m7G cap (a Cap-0 structure to be precise; the Cap-0 structure can be enable translation in mammalian cells and acts similar to the mammalian Cap-1, albeit functions at lower efficiency) and poly(A) tail, facilitating its assembly into VLPs that can serve as effective mRNA delivery vehicles^19^. The assembly process of PVX is characterized by a highly conserved nucleation mechanism, initiating the formation of a stable core structure around its gRNA^20^. This core acts as a foundation for the sequential recruitment and assembly of CP subunits, driven by interactions with specific RNA sequences and structural motifs within the PVX genome^20^. The AC-rich single-stranded sequence and the stem-loop structure at the 5’ region of the PVX genome, called stem-loop 1 (SL1), collectively form the origin of assembly (OAS) site and play pivotal roles in this process, enabling the specific recognition and binding of CP subunits. This intricate assembly mechanism could be harnessed for therapeutic RNA delivery^7^.

Only a few groups have investigated PVX as a nanotechnology platform; principally, we have demonstrated small molecule drug delivery^21^, while others have established protein display for catalysis^22,23^, bone tissue engineering^24^, and epitope display for vaccine applications^25^. The development of PVX for nucleic acid delivery has not been reported. We hypothesized that the positively charged, flexible filamentous protein nanotechnology confers a suitable nanotechnology platform and would enable cell uptake and transduction. Moreover, given the filamentous, flexible nature we proposed the formulation of halo-shaped nanotechnology for circRNA delivery.

Other advantageous properties of PVX are its manufacturing through scalable plant molecular farming or bacterial fermentation offering cost-effectiveness, and environmental sustainability^27^. By implementing molecular farming, fermentation, as well as cell-free synthesis, the production of mRNAs, circRNAs, and PVX CPs could be optimized for distributed manufacturing models closer to treatment sites^27,28^. This strategy has the potential to alleviate supply chain bottlenecks, reduce production costs, and facilitate the global accessibility of mRNA-based therapeutics and vaccines^27,28^.

In this work, we describe the methods to produce PVX loaded with custom mRNA and circRNA and the application thereof. We developed a modular system enabling size, shape, and functional control, the latter enabled through mixed assembly with functionalized CPs. CPs were obtained either from PVX farmed in *Nicotiana benthamiana* plants or recombinant expression in *Escherichia coli*. Packaging of custom cargo was enabled through integration of the PVX OAS site (SL1). Resulting PVX vectors’ morphology and size were characterized through transmission electron microscopy (TEM) and size exclusion chromatography (SEC). Using a fluorescent reporter gene (enhanced green fluorescent protein, EGFP), gene delivery and protein expression was confirmed in mammalian cells using quantitative RT-PCR, flow cytometry, and imaging studies of cultured cell lines.

## Materials and methods

### Molecular cloning

The plasmids used in this study included pCMV-T7-EGFP (BPK1098), a gift from Benjamin Kleinstiver and Harvard University (Addgene plasmid # 133962; http://n2t.net/addgene:133962; RRID: Addgene_133962) and Circ-oc-EMCV-GFP, a gift from Prashant Mali and UC San Diego (Addgene plasmid # 226261; http://n2t.net/addgene:226261; RRID: Addgene_226261). PVX genome plasmids for size control and mixed assembly studies were adapted from pCMV-T7 to include the full PVX genome (pCMV-T7-PVX-Genome; Schematic 1 A). To promote PVX coat protein (CP) binding and in vitro assembly of PVX VLPs on in vitro transcripts, the origin of assembly site (SL1, 60 nts)^6^ was cloned downstream of the T7 promoter and upstream of the EGFP fluorescent reporter gene using gBlocks (Integrated DNA Technologies, San Diego, CA) and Gibson assembly (Gibson Assembly Master Mix, New England Biolabs, Ipswich, MA) to yield pCMV-T7-SL1-EGFP (Schematic 1B). For circRNA, SL1 was cloned downstream of EGFP and before the WPRE sequence to generate Circ-EMCV-GFP-SL1 (Schematic 1 C). Another plasmid, pHGWA-9xHis-PVX-CP, was cloned to contain the PVX CP downstream of an N-terminal 9xHis-tag and TEV cleavage site (Schematic 1D) for mixed assembly VLPs. A cell line derivative of DH5α (NEB 5-alpha Competent *E. coli*, New England Biolabs, Ipswich, MA) was used for cloning the vector constructs and BL21(DE3) (NEB T7 Competent *E. coli*, New England Biolabs, Ipswich, MA) for protein production; sequences were confirmed through either Sanger sequencing (Eurofins Genomics, Louisville, KY) or Nanopore sequencing (Plasmidsaurus, South San Francisco, CA) of plasmid DNA. The plasmid sequences are provided by Addgene as well as the Supporting Information and the gBlocks are detailed in the Supporting Information (Supplementary Tables S1-2).

**Schematic 1:**
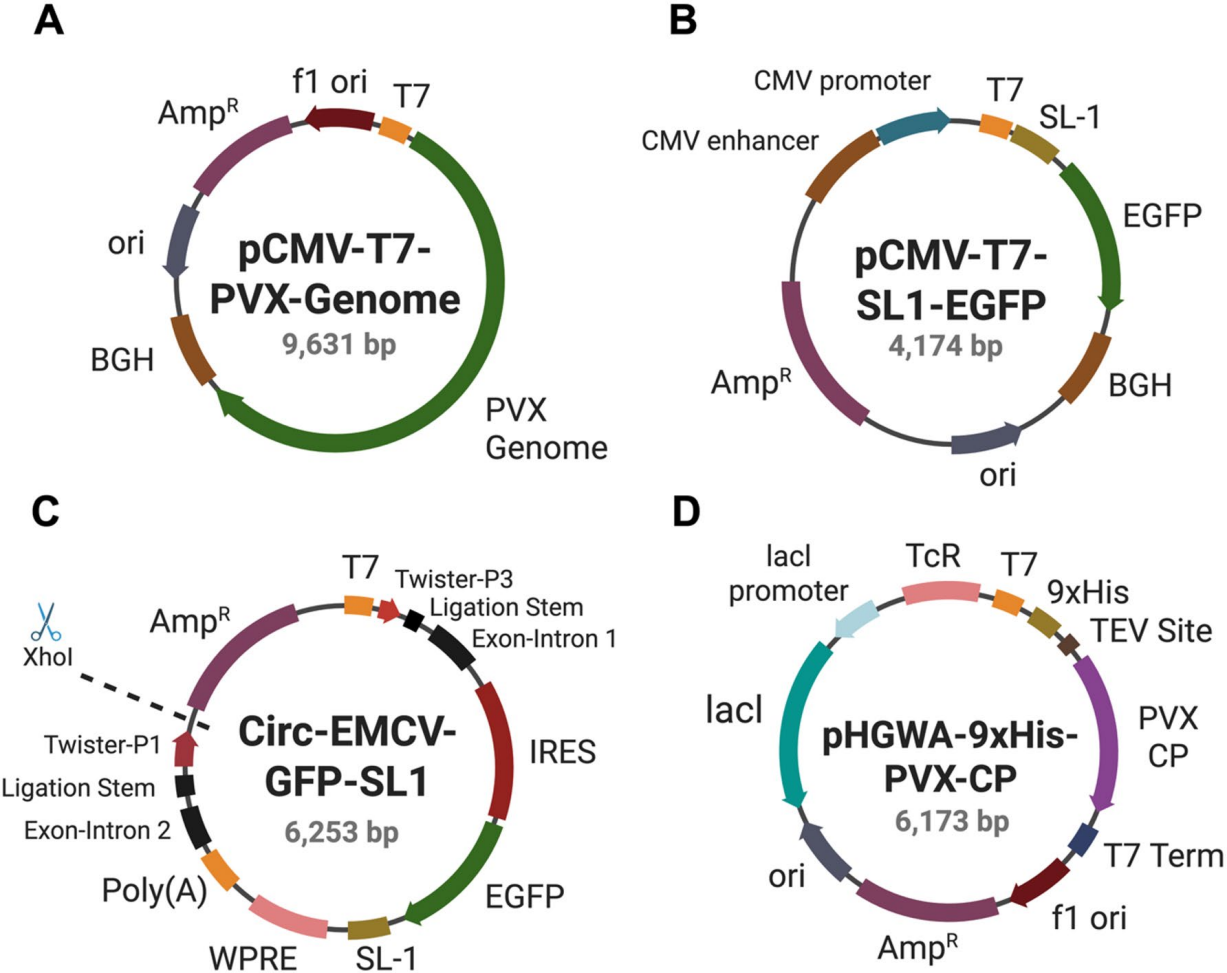
Representation of plasmids used for mRNA production, circRNA synthesis, and PVX CP protein fermentation. (**A**) Plasmid representation of pCMV-T7-PVX-Genome including a promoter for bacteriophage T7 RNA polymerase (T7 promoter), the entire genomic sequence for PVX’s genome (PVX Genome), bovine growth hormone polyadenylation signal (BGH), high-copy-number origin of replication (ori), β-lactamase for resistance to ampicillin/carbenicillin (Amp^R^), and f1 bacteriophage origin of replication for (+) synthesis (f1 ori). (**B**) Map pCMV-T7-SL1-EGFP with features consisting of a promoter for bacteriophage T7 RNA polymerase (T7 promoter), the PVX origin of assembly (SL1), codon-optimized enhanced GFP (EGFP), bovine growth hormone polyadenylation signal (BGH), high-copy-number origin of replication (ori), β-lactamase for resistance to ampicillin/carbenicillin (Amp^R^), and human cytomegalovirus immediate early enhance (CMV enhancer) and promoter (CMV promoter).(**C**) Vector map of Circ-EMCV-EGFP-SL1 including a promoter for bacteriophage T7 RNA polymerase (T7 promoter), flanking twister ribozymes (Twister-P3; Twister P1) that rapidly self-cleave and allow for hybridization of ligation stems, exon-intron sequences (Exon-Intron 1; Exon-Intron 2) that allow for autocatalytic intron splicing, internal ribosomal entry site (IRES), codon-optimized enhanced GFP (EGFP), PVX origin of assembly (SL1), woodchuck hepatitis virus posttranscriptional regulatory element (WPRE), Poly(A), and β-lactamase for resistance to ampicillin/carbenicillin (Amp^R^). (**D**) Plasmid map of pHGWA-9xHis-PVX-CP featuring a promoter for bacteriophage T7 RNA polymerase (T7 promoter), a 9x His affinity tag (9xHis), TEV protease cleavage site (TEV Site), PVX CP, transcription terminator for bacteriophage T7 RNA polymerase (T7 Term), f1 bacteriphage origin of replication for (+) synthesis (f1 ori), β-lactamase for resistance to ampicillin/carbenicillin (Amp^R^), high-copy-number origin of replication (ori), lac repressor (lacl) and its promoter (lacl promoter) for repressing transcription, and tetracycline efflux protein (TcR). Created with BioRender. See also Supporting Information (Supplementary Tables S1 + S2) for details on the gBlocks and plasmid sequences.

### In vitro transcription (IVT)

DNA templates for RNA products were created through PCR amplification (Q5^®^ High-Fidelity 2X Master Mix, New England Biolabs, Ipswich, MA) from plasmid PCR products then amplified using the T7 promoter and BGH poly(A) sites for the forward and reverse primers (Integrated DNA Technologies, San Diego, CA) respectively. Plasmids and designs for circRNA included additional features such as 5’ ribozyme sequence, a 5’ ligation sequence, an IRES sequence linked to the product of interest, a 3’ UTR sequence, a 3’ ligation sequence, a 3’ ribozyme sequence, and a poly-T stretch to terminate transcription. PCR reaction mixtures were treated with DpnI (1 U/µl, New England Biolabs, Ipswich, MA) to degrade template pDNA and PCR products were subsequently purified using a QIAquick PCR Purification Kit (Qiagen, Valencia, CA). The PCR products were then used to generate pre-mRNA using T7 High Yield RNA Synthesis Kits (New England Biolabs, Ipswich, MA) per the manufacturer’s specifications. In vitro transcription (IVT) was carried out in a heat block for 1–2 h at 37 °C. IVT pre-mRNA products were then purified using LiCl precipitation. To complete the processing of pre-mRNA into mature linear mRNAs, a 7-methylguanylate cap (Cap-0) structure was added to the 5′ end of the mRNA using the Vaccinia Virus Capping System (New England Biolabs, Ipswich, MA) and then purified using LiCl before poly(A) tailing using *E. coli* Poly(A) Polymerase (New England Biolabs, Ipswich, MA) according to the vendor’s protocols. The final mRNA was then purified using LiCl precipitation once more, followed by quantification by UV/Vis Spectroscopy (Nanodrop 2000, Thermo Fisher, USA) to measure purity (RNA with a A260/A280 ratio between 2.0 and 2.2 was for experimentation exclusively). Characterization and imaging of mRNA and circRNA sizes were confirmed by gel electrophoresis using a 1.2% (w/v) agarose gel in electrophoresis buffer (1X Tris-Borate-EDTA (TBE) buffer, pH 8.3) and Agilent 2100 bioanalyzer (Agilent, Santa Clara, CA) RNA electropherograms. mRNA was stored at either − 20 °C or -80 °C after IVT and between uses.

In regard to the IVT protocol for circRNA, plasmids containing the RNA template were linearized at the 3’ end of the template sequence using XhoI (New England Biolabs, Ipswich, MA) digestions; the linearized product was then purified using the QiaQuick PCR spin columns. RNA products were then produced using these linearized plasmid templates using the HiScribe T7 Quick High Yield RNA Synthesis Kit (NEB E2040) per the manufacturer’s protocol. IVT RNA reactions were cleaned with the Monarch RNA Cleanup Kit (500 µg) (T2050) according to manufacturer’s instructions. CIP treatment was then performed to ensure the removal of any remaining triphosphates from IVT, including cleaved twister ribozyme product. Cellulose chromatography was performed twice according to Baiersdörfer, M. et al. per 100 µg of RNA^29^. To isolate circular RNA products, 20 µg of circRNA was diluted to a total volume of 88 µL then heated at 65 °C for 5 min and subsequently cooled on ice before adding 30U RNase R (2 µL) and 10 µL of 10X RNase R buffer (MCLAB) and the reaction incubated at 37 °C for 30 min. The reaction was then cleaned again with the Monarch RNA Cleanup Kit (500 µg) (T2050) according to manufacturer’s instructions.

### Preparation of PVX CPs and RNA

PVX virions were propagated and purified from infected *Nicotiana benthamiana* plants as previously described^18^. To isolate CP, a 5 mg suspension of purified PVX in potassium phosphate buffer (0.1 M KP, pH 7.0) was aliquoted into a 10 kDa MWCO Slide-A-Lyzer Dialysis Cassette (Thermo Fisher Scientific, Waltham, MA) and dialyzed against a ‘disassembly buffer’: 50 mM Tris-Cl, pH 7, 750 mM CaCl_2_, 1 mM EDTA, 1 mM DTT, and 0.5 mM PMSF for 8–24 h at 4 °C to disassemble the virions. Dissociated suspensions were then centrifuged at 20,000 g for 20 min at 4 °C to remove gRNA, followed by ultracentrifugation at 180,000 g for 1 h and 10 min at 4 °C to remove any intact virions. gRNA pellets were resuspended in nuclease-free water and precipitated using LiCl precipitation. PVX CP suspensions were then dialyzed against an ‘assembly buffer’ containing 10 mM Tris-HCl, pH 8.0, and 50 mM NaCl for 8–24 h at 4 °C. This was followed by centrifugation at 20,000 g for 20 min at 4 °C to remove any residual gRNA, followed by ultracentrifugation at 180,000 g for 1 h and 10 min at 4 °C to remove any reassembled VLPs. The CP was concentrated using Amicon 10 kDa MWCO Ultra Centrifugal Filter tubes (Millipore Sigma, Burlington, MA) and the concentration was determined through a Pierce BCA Protein assay (Thermo Fisher Scientific, Waltham, MA). The integrity of the CP and the removal of residual gRNA were confirmed by size exclusion chromatography, chromatography ÄKTA Explorer chromatography system with a Superose 6 Increase column (GE Healthcare, Chicago, IL); the A260:280 nm ratio of pure CP is ≤ 0.57.

### In vitro assembly of PVX VLPs with mRNA, circRNA, or PVX gRNA

Assembly reactions with in vitro transcribed EGFP-SL1 mRNAs, genomic fragment mRNAs, Circ-EGFP-SL1, and PVX gRNA were carried out at room temperature using a 50:1 CP: RNA mass ratio using assembly buffer for 3–24 h at concentrations between 1 and 5 mg/mL (Schematic 2). Assembled VLPs were then centrifuged at 20,000 g for 20 min at 4 °C to remove any residual RNA. Each in vitro assembled VLP preparation was purified through a sucrose cushion gradient (30% in Tris-buffered saline [TBS] – 150 mM NaCl, 50 mM Tris-HCl, pH 7.6) and ultracentrifuged at 180,000 g for 1 h and 10 min at 4 °C. VLP pellets were resuspended in either assembly or potassium phosphate buffer (0.01 M KP, pH 7.0) with 5–10% sucrose (w/v).

### Cloning and in vitro assembly of Size-Controlled VLPs

**Fig. 1:**
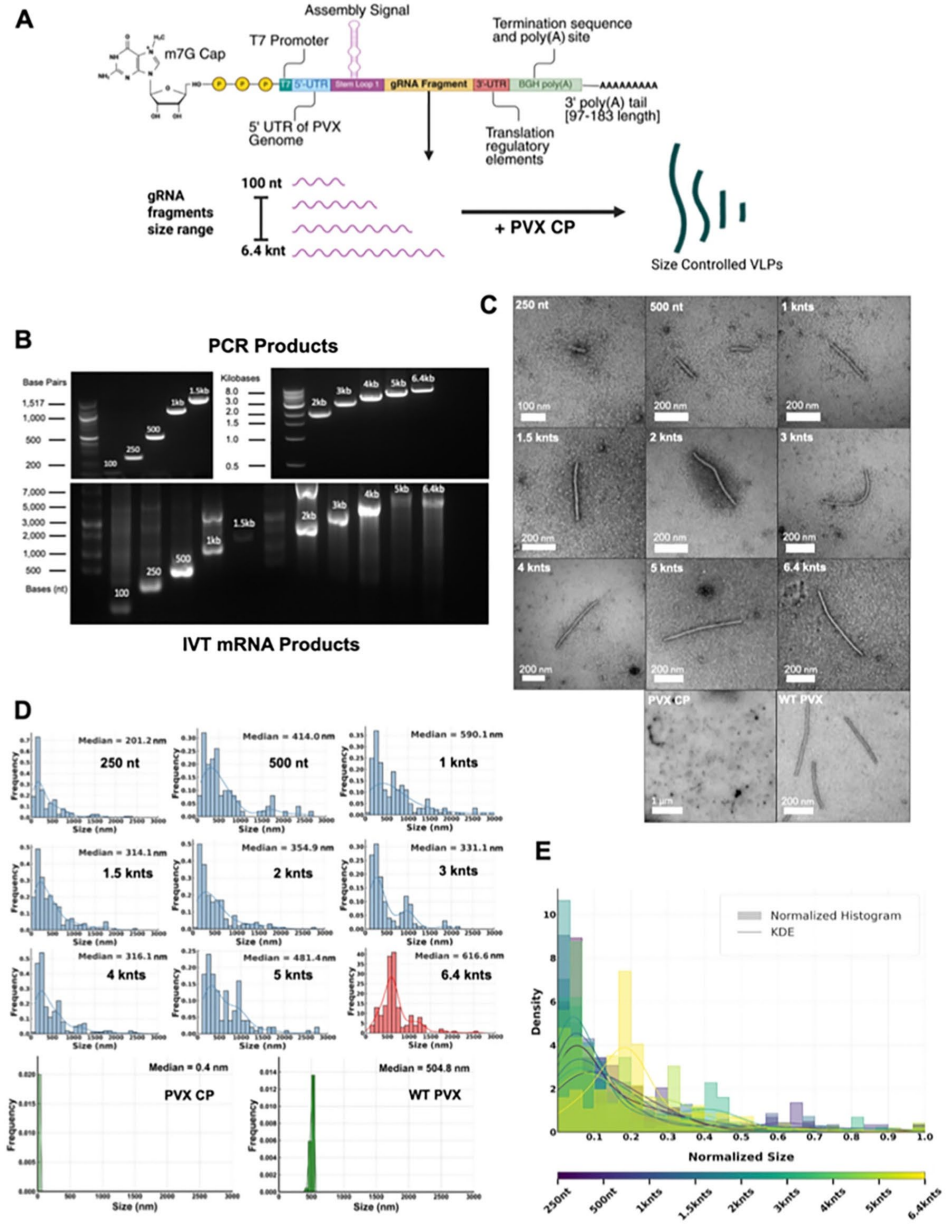
Size control of PVX VLPs through templated assembly of CP on (gRNA) fragments. (**A**) Graphical representation of genomic mRNA fragment design and VLP production. Created with BioRender. (**B**) Agarose gel electrophoresis of PCR products (upper) and gRNA fragments after m7G capping, poly(A) tailing, and purification (lower). Uncropped images are provided in Supporting Figure S1. (**C**) TEM images of representative VLPs, CPs, and PVX. (**D**) Histograms normalized to most frequent VLP sizes per gRNA length as compared to the negative control (PVX CP) and positive control (WT PVX); the 6.4-kb assembly is colored red to show a significant size distribution shift and to highlight which VLP condition exhibits the largest size shift. (**E**) Size distributions of measured particles from TEM images across different size conditions with density line overlays; colored as a spectrum ranging from smallest (250 nt, purple) to largest conditions (6.4 kb, yellow). VLP lengths (nm) were normalized to each dataset’s maximum value to compare relative distribution shapes across conditions with different input RNA sizes. Histograms show the normalized frequency of particle lengths, and Kernal Density Estimation (KDE) curves provide smoothed density estimates). KDE is a non-parametric method for estimating the probability density function of a dataset by summing smooth kernel functions (e.g., Gaussian) centered at each observation, producing a continuous curve that represents the underlying distribution.

In vitro transcribed size-controlled mRNA transcripts, derived from gRNA sequences cloned into the pCMV-T7-PVX-Genome plasmid, were designed as shown in Fig. 1A. The constructs include a T7 promoter, the PVX genome, and a BGH poly(A) site. The PVX origin of assembly site (OAS) sequence consists of a *cis-*acting element from the 5’ region of PVX gRNA that includes an AC-rich single-stranded region from nt 1–49 and downstream SL1 located at nt 49–85, which forms the OAS. To produce PVX VLPs of distinct sizes, vectors were designed with the full-length PVX RNA genome of 6.4 kb and smaller genomic fragments (shortened at the 3’ end) of 5 kb, 4 kb, 3 kb, 2 kb, 1.5 kb, 1 kb, 500 bases, 250 bases and 100 bases (Fig. 1A). These were then in vitro transcribed using a T7 RNA polymerase transcription kit, capped with a 7-methylguanylate cap (m7G; Cap-0), and poly(A) tailed. Following IVT, products were LiCl purified and characterized using 1.2% (w/v) agarose gels in TBE (Fig. 1B), and template plasmids were confirmed through either Sanger or Nanopore sequencing.

**Schematic 2:**
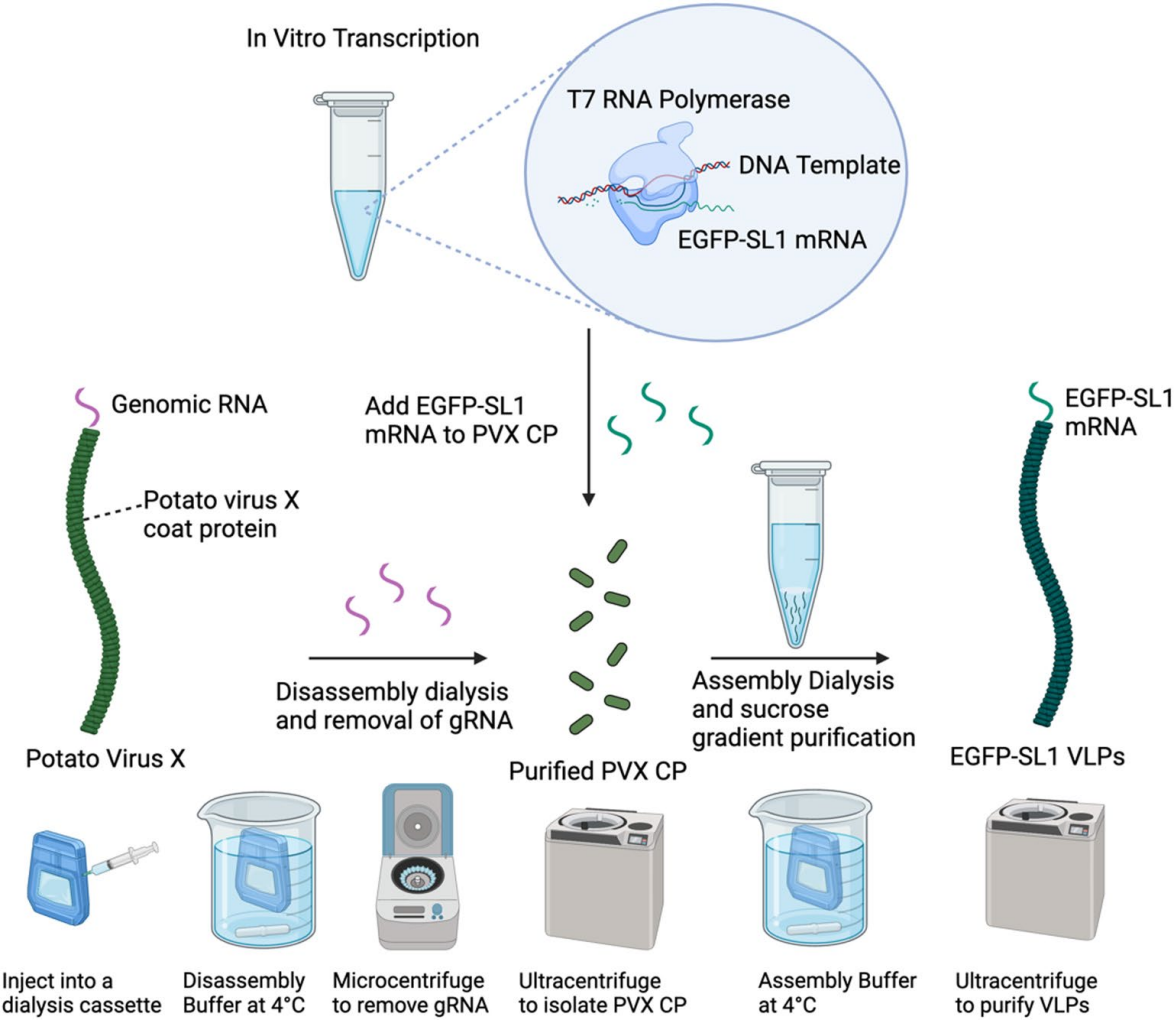
Representation of in vitro assembly process for PVX VLPs and in vitro transcription of mRNA transcripts using the EGFP-SL1 payload as an example. Created with BioRender.

### Characterization of assembled VLPs

VLP and PVX morphology and structure were examined with a JEOL 1400 plus transmission electron microscope (TEM) (CMM Electron Microscopy Facility, San Diego, CA) at 80 kV with a Gatan OneView 4k x 4k camera. VLPs and PVX (each at 0.1 mg/ml) were prepared for TEM using glow-discharged carbon paper-coated copper (200-mesh) PELCO Pinpointer grids (Ted Pella, USA). After a 2 min sample deposition step (repeated twice), the grids were blotted on Whatman filter paper and washed twice with nuclease-free water (1 min each) before negative staining with 2% (w/v) uranyl acetate (10 µl) for 30 s. Additional steps were performed for gold nanoparticle-conjugated VLPs as described below. Images were captured at 15kX magnification and processed using Fiji biological-image analysis software (v2.9) to determine VLP length and diameter through pixel-to-nanometer conversions. Purity and size distribution profiles of VLPs were determined through their elution profiles on an ÄKTA Explorer chromatography system with a Superose 6 Increase column (GE Healthcare, Chicago, IL, USA). VLPs were run in assembly buffer (10 mM Tris-HCl, pH 8.0, and 50 mM NaCl) at a concentration of 0.1 mg/mL at full column volume at a flow rate of 0.5 mL/min.

### Characterization of mixed VLP assemblies by Immunogold TEM and Western blot

For mixed VLP assemblies containing a ratio of recombinant (His-tagged): wildtype CPs, 10 nm Ni-NTA-Nanogold^®^ (Nanoprobes, Yaphank, NY), diluted 1/30 in nuclease-free water, was added to the grid after sample deposition (see above) followed by a 30-minute incubation at room temperature and a 1 min wash with 20 mM Tris at pH 7.6 with 150 NaCl containing 8 mM imidazole. Additionally, mixed VLP assemblies were characterized using Goldiblot™ His-tag Western Blot Kits (Nanoprobes, Yaphank, NY). CPs were separated by 4–12% SDS-PAGE and then transferred to PVDF or nitrocellulose membranes. Membranes were then equilibrated with 20 mM Tris, 0.15 M NaCl, pH 7.6, 0.1% (w/v) Tween^®^-20 (TBS-0.1%T) for 3 min, blocked with 5% (w/v) nonfat dry milk in TBS-0.1%T for 15 min, and then incubated with 0.1 ml of Nickel-NTA-Nanogold^®^ added to 10 mL of 1% (w/v) nonfat dry milk in 20 mM Tris, 0.15 M NaCl pH 7.6, 0.6% (w/v) Tween^®^-20 (TBS-0.6%T) for 30 min, followed by washing the membrane twice with 15 mL of 10 mM imidazole in TBS-0.6%T for 2 min each, washing the membrane three times with deionized water for 3 min each, simultaneously mixing the Goldiblot™ AutoMet Detect ABCD mix, adding ABCD mix to sample for 6–20 min, and washing three times with deionized water for 3 min each time before drying. After fully dried, membranes were imaged using a FluoroChem R System (ProteinSimple, San Jose, CA, USA) using the epi-white illumination setting.

### Transfection of mRNA, circRNA, and VLPs

Free EGFP-SL1 mRNA, RNase A-treated mRNA, mRNA-containing VLPs, and RNase A-treated VLPs were used to transfect BHK-21 cells (ATCC, Manassas, VA). 12-well plates were seeded with 100,000 cells/well and 1 ml Dulbecco’s Modified Eagle Medium (DMEM, Thermo Fisher Scientific, Waltham, MA) supplemented with 10% (v/v) fetal bovine serum (FBS, Thermo Fisher Scientific, Waltham, MA) and 1% (w/v) penicillin/streptomycin (Thermo Fisher Scientific, Waltham, MA) per well the day before transfection to reach 60–80% confluency. BHK-21 cells were then transfected using 1–2 µg/well of mRNA or 100–300 µg/well VLPs and 5–12.5 µl of Lipofectamine 2000 (Thermo Fisher Scientific, Waltham, MA) according to the manufacturer’s guidelines and specifications. circRNA controls and VLPs were treated similarly for HEK-293T cells plated onto 12-well plates at 100,000 cells/well. VLPs were first diluted into a 20 mM HEPES solution for 10 min before forming complexes with Lipofectamine in Opti-MEM (Thermo Fisher Scientific, Waltham, MA). For RNase-treated conditions, RNase A (PureLink RNase A; Thermo Fisher Scientific, Waltham, MA) digestion was performed at a ratio of 2 ng RNase A to 1 µg VLPs and 0.5 ng RNase A to 1 µg RNA for 1 h at 4 °C before encapsulation. RNase digestion was terminated through the addition of a 1:1 ratio of Ribolock RNase Inhibitor (Thermo Fisher Scientific, Waltham, MA).

### Quantitative RT-PCR of disassembled VLP mRNAs

To confirm the ability of VLPs to encapsulate and protect EGFP-SL1 mRNA, VLP and control suspensions were dialyzed overnight in disassembly buffer followed by centrifugation at 20,000 g at 4 °C for 20 min to pellet the mRNA. The pellets were then resuspended in nuclease-free water and precipitated using LiCl. Following mRNA purification, cDNA synthesis was performed using an RT^2^ First Strand Kit (Qiagen, Valencia, CA). The quantitative RT-PCR assay was then executed using RT^2^ SYBR^®^ Green qPCR Mastermixes (Qiagen, Valencia, CA) along with transgene specific primers (T7 forward 5’ –taatacgactcactatagggagagc – 3’, BGH 5’ – TAGAAGGCACGTCGAGGC – 3’). EGFP-SL1 mRNA was used as a positive control in addition to PVX virions, gRNA VLPs, and CP as negative controls. EGFP mRNA was used as the relative expression internal control gene and real-time efficiencies (E) were calculated using the crossing point difference (CPΔ). The RT-PCR assay was executed on a Biorad CFX96 Real-Time System thermal cycler (Biorad, Hercules, CA). Each assay sample set was performed in triplicate.

### Flow cytometry

Transfected BHK-21 and HEK 293T cell lines were harvested using Gibco Trypsin-EDTA (0.05% [w/v]) (Thermo Fisher Scientific, Waltham, MA), followed by centrifugation at 500 g, washing with 1X PBS, pH 7.4, fixation with 4% (w/v) paraformaldehyde in PBS for 10 min, and resuspension in 1X PBS, pH 7.4, containing 5% (v/v) FBS. The resuspended cells were then plated in a 96-well plate in triplicate and immediately analyzed for fluorescence using a BD Accuri C6 Plus flow cytometer. Flow cytometry results were analyzed using FlowJo v10 software as specified previously. Each transfection experiment was performed in biological triplicates.

### Confocal microscopy

Cells were prepared for confocal microscopy by plating them on either an 8-chamber well slide (Millicel EZ Slide, Millipore Sigma, Burlington, MA) or 6-well plate with coverslip 16–24 h before transfection. 8-chamber slides and coverslips removed from 6-well plates were mounted with Fluoroshield™ (Sigma-Aldrich, St. Louis, MO, USA). At timepoints of 0, 24, 48, and 72 h post-transfections, cell media was aspirated, wells were washed with 1X PBS, pH 7.4 and then fixed using 4% (w/v) paraformaldehyde in 1X PBS, pH 7.4 and resuspended in 1X PBS, pH 7.4 for imaging. Confocal imaging was carried out using a Nikon A1R TIRF STORM Confocal microscope courtesy of the Cancer Center Microscopy Shared Resource at UC San Diego’s Moore’s Cancer Center.

## Results

### PVX VLPs can encapsulate RNA cargos of different lengths

In vitro transcribed size-controlled mRNA transcripts, derived from PVX genomic RNA (gRNA) fragments cloned into the pCMV-T7-PVX-Genome plasmid, were designed as shown in Fig. 1A. gRNA fragments ranging from sizes of 100 bp-6.4 kb were generated by PCR amplification from the plasmid followed by IVT to yield pre-mRNA that was then poly(A)-tailed and capped. IVT of the 100 nt-long RNAs was low yielding (Fig. 1B). It was also apparent that, especially for the shorter RNAs, higher molecular weight bands were detected, resulting in a laddering effect in the size range of 1–5 kb – it is these contaminants that may have caused formation of PVX assemblies longer than predicted (see below).

For size-controlled VLP assembly and packaging of mRNA containing various lengths of gRNA fragments, purified CPs from PVX purified from *N. benthamiana* were used; re-assembly reactions were carried out using a 50:1 mass ratio of CP: RNA (or 4,200 CPs per mRNA molecule)^7^. The size distribution of the assembled VLPs was then determined from TEM images (Fig. 1C) taken at magnifications of 15kX to 30kX and quantified through standardized pixel-to-nanometer measurements using FIJI 2.9’s image processing packages, using a minimum of 50 images and 130 particles per condition. This was plotted as histogram distributions of VLP measurements by frequency of size measurements that displayed a trend in increasing size by input mRNA size, and a sizable distribution shift when presented with mRNA of the same size as PVX’s native gRNA (Fig. 1D and E). Representative TEM images of each median VLP size displayed the characteristic filamentous morphology when packaging ≥1-kb mRNA templates; in contrast VLPs assembled on shorter templates, 250 nt and 500 nt mRNA, resembled more rigid rod-shaped structures (Fig. 1C).

When analyzing the size of the assembled VLPs containing mRNA cargos of various lengths, trends can be observed that implicate viral mechanisms of in vitro assembly that contrast with evolved in vivo assembly. PVX VLPs, when packaging native PVX gRNA, were measured at a median length of 616.6 nm – longer than the measured median length of native PVX virions at 504.8 nm (as shown in Fig. 1C-E) – despite the formation of particles using the same starting materials (wildtype CP and gRNA). This is likely due to less optimal RNA-packaging during in vitro assembly, leading to decreased packaging efficiency and stability of the VLP or nucleation of RNA-CP binding events that create hollow “tube ends” beyond the length of the packaged RNA. In principle template RNA should serve as a guide and constraint for VLP size, however when observing the other size conditions in our studies we could only note a trend for size-control. For example, VLPs packaging mRNA of 3-kb measured about half in length (median length: 331 nm) compared to VLPs with the native genome – this is as predicted. However, VLPs packaging mRNA 1.5 kb in length also displayed a median length of 314 nm. These discrepancies in size may be explained by the contamination of assembly reactions with larger RNA fragments (as observed by agarose gel electrophoresis, Fig. 1B), end-to-end or overlapping alignments of smaller genomic fragment mRNAs forming multiplets, and overextension of the CP nucleation mechanism leading to empty ends with genomic mRNA template.

### PVX VLPs can assemble with mixed wildtype and His-tagged CPs

To streamline manufacture of the CP, we also produced the PVX CP in *E. coli* to complement the more specialized plant molecular farming with a more widely established technique. At the same time, we introduced a His-tag into the CP to enable purification but also to introduce a handle for functionalization: the His-tag allows for functionalization through coordination with Ni-NTA-appended cargos. In the future such system could be used to display targeting ligands (peptides, antibodies, aptamers) to achieve tissue-specificity.

Recombinant CP-His (rCP-His) was produced at yields ranging from 204 to 265.5 mg per 1 L bacterial culture (Supplemental S2A). While rCP-His protein alone did not yield VLPs when templated on gRNA – the addition of native CP enabled formation of VLPs containing a mixture of rCP-His and native CP. We note that both, rCP-His with N-terminal or C-terminal His-tags could be expressed in and purified from *E. coli*, however the C-terminal His-tag – with or without the addition of native CP – did not yield VLPs (Supplemental 2B-C); therefore N-terminal rCP-His should be considered.

**Fig. 2:**
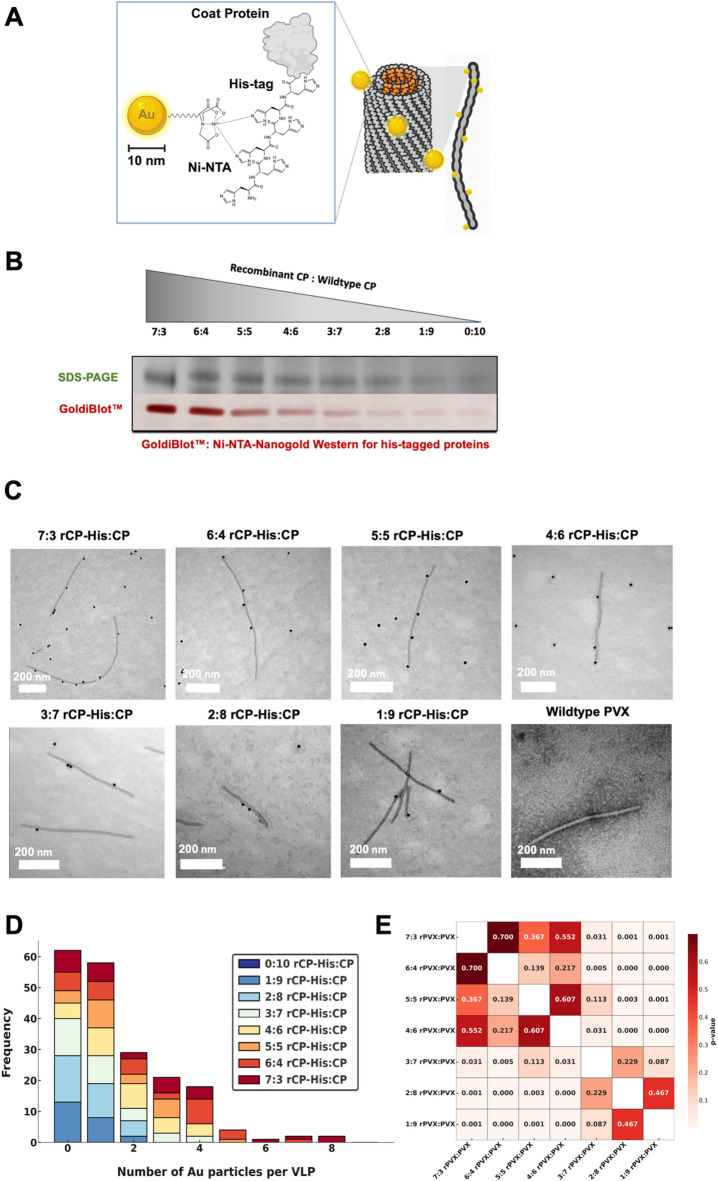
VLPs were obtained through mixing defined ratios of recombinant rCP-His and wildtype CPs. (**A**) Representative cartoon of the mechanisms for 10 nm Ni-NTA-Nanogold^®^ conjugation to the N-terminal 9xHis-tag on recombinant PVX CPs derived from *E. coli* fermentation (created using BioRender). (**B**) 4–12% SDS-Page (upper) and Goldiblot™ western (lower) of mixed assembly VLPs organized by CP ratios. Uncropped gels and blots are shown in Supplemental Figure S2C. (**C**) Representative TEM images of Au 10 nm Ni-NTA-Nanogold^®^-conjugated mixed assembly VLPs. (**D**) Measured frequencies, as measured by the number of Au particles bound to VLPs organized by mixed assembly ratio condition. (**E**) Pairwise statistical comparison of bound Au particle frequencies using the Mann-Whitney U test. The heatmap displays the p-values from nonparametric comparisons between all groups, with lower p-values (lighter cells) indicating greater statistical significance in Au nanoparticle-VLP conjugation frequencies by ratio combination.

Native CP (obtained from PVX produced in plants, purified, and then disassembled) and rCP-His were mixed at varying ratios 7:3 − 0:10 rCP-His: CP; then mixed with gRNA purified from native PVX; the CP: RNA mass ratio was kept at 50:1. Mixed assembly reactions yielded VLPs with fidelity using ratios ranging from 7:3 rCP-His: CP to 1:9 rCP-His: CP, whereas ratios of 8:2, 9:1, and 10:0 rCP-His: CP were unable to reliably produce VLPs (Supplemental S2D). This indicates that a certain degree of native CP is required to enable nucleoprotein assembly. To confirm the incorporation of rCP-His into the mixed assembly VLPs, 10 nm Ni-NTA-Nanogold^®^ gold nanoparticles were used for detection in a Goldiblot™ western blot assay and immunogold TEM imaging (Fig. 2B-C). Goldiblot™ western results revealed increasing band intensity with VLP samples that were assembled from higher rCP-His: CP ratios, indicating that the addition of higher equivalents of rCP-His leads to higher incorporation of the rCP-His protein (Fig. 2B and Figure S2E). The presence of rCP-His and the ability to functionalize the nucleoprotein assemblies via the tag was further validated using Nanogold^®^ TEM (Fig. 2C). It should be noted that this method is not quantitative but serves to analyze trends. 130–210 VLPs per assembly condition were imaged at 15kX to 60kX and analyzed using FIJI 2.9’s image processing packages, then plotted as a stacked bar chart (Fig. 2D). Data analysis indicated a trend: increased rCP-His added into the assembly mix correlated with a larger number of gold nanoparticles bound per VLP – thus data is in agreement with the western blot results. At the lower end of 1:9 and 2:8 rCP-His: CP VLPs displayed up to 2 gold nanoparticles only; at the 3:7 and 4:6 ratio up to 4 gold nanoparticles per VLP were counted; counts continued to increase with the 5:5 condition yielding up to 5 gold nanoparticles per VLP, 6:4 resulted in up to 7 gold nanoparticles per VLP, and with the 7:3 condition up to 8 gold nanoparticles per VLP were observed (Fig. 2D). To assess differences between the frequencies of gold nanoparticle per VLPs as a function of rCP-His: CP ratios, a Mann-Whiteney U Test was performed and visualized using a heatmap colorized by p-values (Fig. 2E). Strong statistical significance (*p* < 0.001–0.1) was observed between the lower rCP-His: CP ratios as compared to the higher rCP-His: CP ratios, with lower significance observed between neighboring or similar conditions.

### PVX VLPs are capable of packaging foreign IVT mRNAs for delivery and protein expression in mammalian cells

**Fig. 3:**
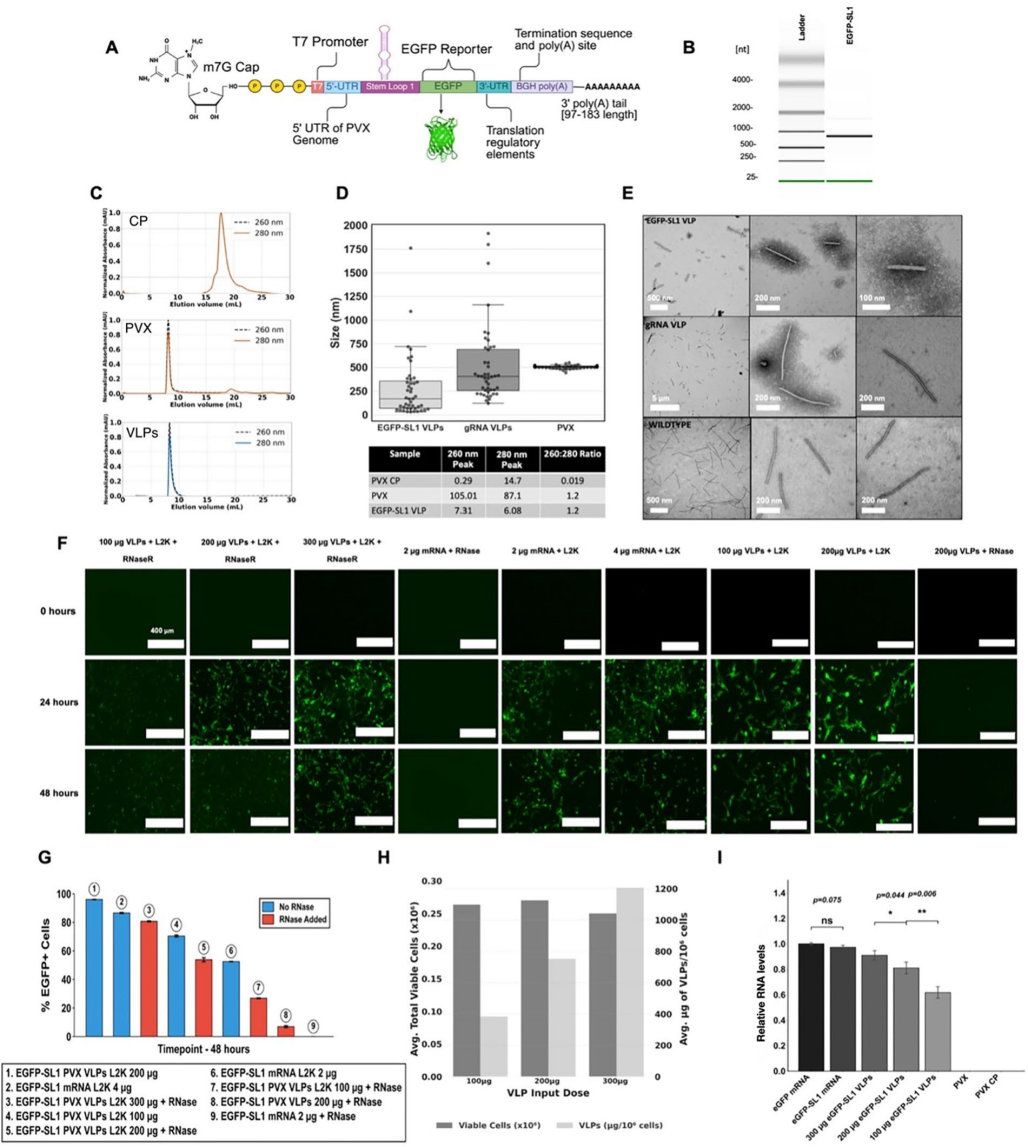
Design and analysis of EGFP-SL1 VLPs. (**A**) Schematic representation of the mRNA cassette containing EGFP-SL1. EGFP-SL1 mRNA was constructed by encoding the OAS site of the PVX genome, stem-loop 1 (SL1), upstream of the EGFP reporter sequence, flanked by the untranslated regions at both the 5’ and 3’ terminus, and joined with an m7G cap at the 5’ terminal end and a Poly(A) tail at the 3’ terminal end. (**B**) mRNA transcripts generated by in vitro transcription (IVT) and purified by LiCl precipitation were quantified and analyzed in comparison with the heat-denatured Agilent RNA 6000 ladder using the Agilent 2100 bioanalyzer system. (**C**) Size exclusion chromatograms (SEC) of PVX CP control (top), native PVX (middle) and VLPs assembled with EGFP-SL1 mRNA (bottom). The size distribution of measured particles of in vitro assembled EGFP-SL1 VLPs (blue and black) as compared to VLPs made with PVX CP (orange) and PVX gRNA (orange and black). (**D**) Length measurements from TEM measurements (upper) and sample 260 nm:280 nm ratios of EGFP-SL1 VLPs as compared to PVX and PVX CP (lower). (**E**) TEM micrographs of VLPs made with EGFP-SL1 (upper), gRNA (middle), and wildtype PVX (lower). (**F**) Confocal microscopy of BHK-21 cells transfected with different conditions of VLPs and mRNA (listed); scale bar represents 400 μm. (**G**) Transfection efficiencies of the corresponding conditions as measured by % EGFP + cells. (**H**) Distribution of mass of VLPs, in µg, per million viable cells in triplicate. (**I**) RT-PCR showing relative RNA levels of free EGFP-SL1 mRNA, disassembled EGFP-SL1 VLPs (100–300 µg), free CP, and disassembled PVX virus in relation to original EGFP mRNA (no SL1). Error bars represent standard deviation. Statistical significance was calculated through two-way ANOVA.; *** *p* < 0.0001, ** *p* < 0.005, * *p* < 0.05, NS: not significant. Abbreviations: L2K = Lipofectamine 2,000, EGFP = enhanced green fluorescent protein, PVX = potato virus X, CP = coat protein, and VLP = virus-like particle.

To assemble VLPs and package mRNA payloads for protein expression, CPs were isolated from PVX produced in plants and reassembled with either target mRNA or gRNA at a mass 50:1 ratio of CP: RNA. Figure 3A depicts the mRNA coding for the reporter gene EGFP – the purity and size of the mRNA construct was confirmed by analysis using an Agilent 2100 bioanalyzer system (Fig. 3B).

SEC was used to determine the purity, size profile, and RNA encapsulation of VLPs in comparison to free CP and native PVX. Free PVX CP had a A260nm:280 nm ratio of 0.02 while PVX and assembled EGFP-SL1 VLPs had a A260 nm: 280 nm ratio of 1.2 – characteristic of intact nucleoprotein assemblies (Fig. 3C). The elution profile of EGFP-SL1 VLPs was comparable to that of native PVX – the slight shift from 8.27 ml elution volume for native PVX compared to 8.63 ml for EGFP-SL1 is consistent with the shorter length of the EGFP-SL1 vs. native PVX, indicates a significantly smaller particle size (Fig. 3C).

Size distributions of VLPs were also derived from TEM images taken at 15kX magnification and quantified through standardized pixel-to-nanometer measurements using FIJI 2.9’s image processing packages. EGFP-SL1 VLPs displayed an average size of 277.2 nm, a minimum size of 29.6 nm, a maximum size of 1,762 nm, and a median size of 169.3 nm (Fig. 3D). In comparison, PVX gRNA VLPs exhibited an average size of 519.3 nm, a minimum size of 122.2 nm, a maximum size of 1,917 nm, and a median size of 403.9 nm (Fig. 3D), demonstrating a size profile similar to native PVX (~ 485–525 nm; Fig. 3D). EGFP-SL1 VLPs with the 870 nt mRNA, in principle, should measure ~ 70 nm. The increased median size of 169.3 nm may be attributed to gRNA contamination, multiple or overlapping RNA transcripts incorporated into a single VLP, or overextended assembly and size-control experiments need further optimization (as discussed above). The morphology of EGFP-SL1 VLPs exhibited similar filamentous structures as compared to gRNA VLPs and native PVX (Fig. 3E). The diameters remained consistent at ~ 15 nm across all VLP and native PVX groups, while the aspect ratio remained large across all size conditions with the smallest being shorter EGFP-SL1 VLP particles (Fig. 3E). These findings suggest that the properties of assembled VLPs retain the original qualities found in native PVX despite the encapsulation of a foreign mRNA template containing an OAS structure.

The ability of EGFP-SL1 VLPs to deliver mRNA and express EGFP was then assessed through flow cytometry measurements using in BHK-21 cells (Figs. 3F-G). Higher doses of VLPs transfected with Lipofectamine 2,000 exhibited the greatest % EGFP expression. When RNase was added the gene expression efficiency of the free mRNA constructs was greatly diminished – in stark contrast, packaging mRNA into PVX VLPs conferred protection of the mRNA from enzymatic degradation. Without RNase treatment the expression levels of mRNA-laden VLPs vs. free mRNA were comparable (using lipofected and normalized mRNA amounts − 200 µg VLPs contain 4 µg mRNA). However, when RNase treated, the VLPs outperformed (Fig. 3F and G). Finally, when unassisted by Lipofectamine, EGFP-SL1 VLPs exhibit modest and variable delivery efficiencies, which could be further enhanced through genetic and chemical modifications. It is of note, that in vitro plant VLPs have been shown to require lipofectamine, but in vivo lipofectamine is not required^16^. This highlights the differences between in vitro and in vivo testing and will require follow-up studies to delineate these mechanistic differences. The mass of VLPs (in µg) per million cells for each condition was also quantified to showcase the relationship between dose-escalation and cell viability demonstrating low cytotoxicity of treated cells (Fig. 3H). Finally, we assayed the quality of the mRNA packaged in the VLPs: mRNA was isolated from disassembled VLPs and efficiency of cell transfection was assayed compared to free EGFP-SL1 mRNA. mRNA isolated from EGFP-SL1 VLPs was detected at levels comparable to that of free mRNA (Fig. 3I).

### PVX CPs are capable of encapsulating circrna to form halo-shaped VLPs

**Fig. 4:**
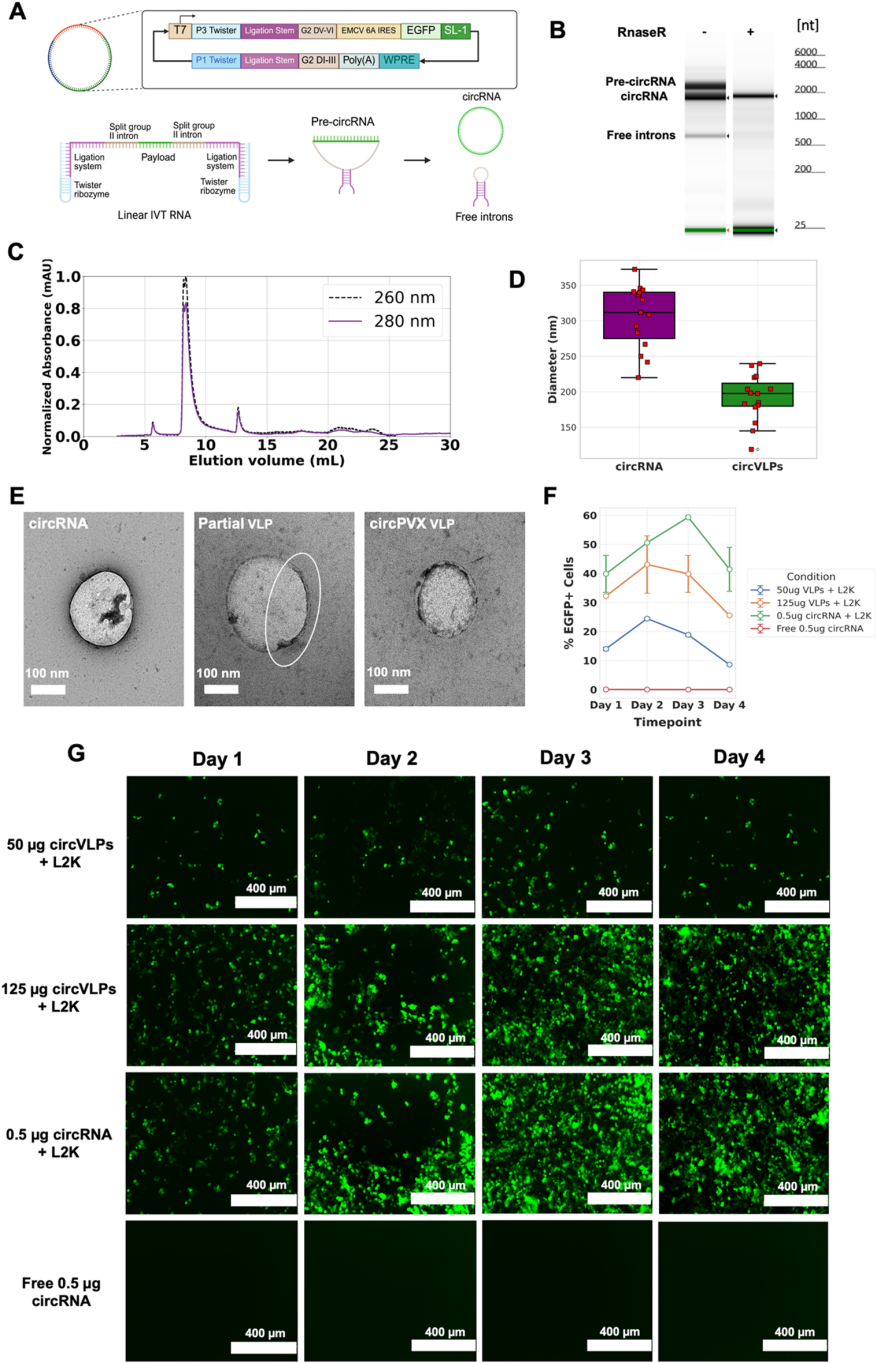
Design, characterization, and analysis of EGFP-SL1 circRNA VLPs. (**A**) Schematic representation of circRNA genetic components, IVT reaction, and circularization mechanism. (**B**) pre-circRNA and circRNA transcripts, as well as free introns, generated by in vitro transcription and purified by LiCl precipitation were analyzed in comparison with the heat-denatured Agilent RNA 6000 ladder before and after the addition of RNAseR. (**C**) Size exclusion chromatogram of EGFP-SL1 circVLPs as overlayed 260 nm and 280 nm distributions. (**D**) Size measurements of circVLPs and circRNA from TEM micrographs. (**E**) TEM micrographs of EGFP-SL1 circRNA, partial circVLP, and full circVLP. (**F**) Transfection delivery efficiencies, as measured by flow cytometry, for EGFP + HEK 293T cells expressing payloads from circVLPs and circRNA controls. (**G**) Confocal microscopy images of transfected HEK 293T cells across the different timepoints and conditions.

To test whether the PVX encapsulation and nucleation mechanism could incorporate other classes of RNA, a circRNA plasmid was adapted from ref^32^. The design, as shown in Fig. 4A – the host plasmid contains several features necessary for producing circRNA. It includes the group II intron from *Clostridium tetani*^33–35^ as previously described, where the I, II, and III domains were split apart from domains V and VI at domain IV to retain properties for intron splicing while allowing for RNA circularization. The domains of the intron were permuted to include domains V and VI at the 5’ end of the construct whereas domains I to III were included at the 3’ end. A minor leader sequence from domain DV/DVI group A was kept since it was likely to include the exon binding site (EBS) necessary for domain I intron-mediated selective splicing. To improved long-range interactions, flanking twister ribozymes that self-cleave at a high rate during IVT and allow for hybridization of complementary ligation stems to each other downstream. These circularization features are next to an internal ribosome entry site (IRES) paired with an EGFP reporter gene and a downstream PVX SL1 sequence before a 3’ UTR.

DNA templates were created through PCR followed by IVT of the RNA and its circularization. Three different sites were chosen to test optimal SL1 placement: post-IRES, post-EGFP, and post-WPRE, with the post-EGFP displaying good circularization rates and expression in HEK 293T cells (Supplemental S3A-C) To characterize circRNAs and confirm circularization, a Bioanalyzer was used to visualize three species: pre-circRNA, circRNA, and free introns (Fig. 4B and Supplemental S3C). Three bands can be observed in the absence of RNaseR, a nuclease that degrades linear RNA, and after treatment with RNaseR a single band representing the purified circRNA can be seen at 2,282 nt. To assemble circVLPs, purified CPs from PVX produced in plants were reassembled in vitro at a mass ratio of 40:1 CP: circRNA. SEC was used to determine the purity, size profile, and RNA encapsulation of VLPs (Fig. 4C). EGFP-SL1 circVLPs displayed a similar A260nm:280 nm ratio of 1.2 to PVX, a tight distribution, and an elution volume of 8.41 ml (which comparable to the elution profile of native PVX, which eluted at 8.21 ml from the Superose6 Increase column); the delayed elution indicates a more compact size of the circVLPs vs. native, linear PVX. While the shape of halo-PVX vs. native PVX is distinct, significant differences in SEC were not apparent, which is explained by the large size of the nucleoprotein assemblies eluting close to the void volume of the size exclusion column.

To calculate the quantitative size distribution of circVLPs, in comparison to circRNA templates, diameter measurements were derived from TEM images taken at 15kX-40kX magnification and quantified through standardized pixel-to-nanometer measurements using FIJI 2.9’s image processing packages (Fig. 4D-E). EGFP-SL1 circVLPs displayed an average diameter of 191.2 nm, a minimum diameter of 118.8 nm, a maximum diameter of 239.6 nm, and a median diameter of 197.58 nm (Fig. 4D). In comparison, circRNAs exhibited an average diameter of 305.53 nm, a minimum diameter of 219.8 nm, a maximum diameter of 372.55 nm, and a median diameter of 311.64 nm (Fig. 4D). Given that the contour length of RNA is 0.34 nm per base; the contour length of the PVX genome is ~ 2,000 nm which is packed into a ~ 500 nm-long nucleoprotein assembly – the condensation factor is 4. The circRNA of 2,282 nt would have a theoretical diameter of ~ 250 nm (measured 305 nm) and considering a condensation factor, the theoretical size of circVLPs would be < 100 nm. Given the size was measured at 200 nm it is fair to assume that packaging of the RNA into the CPs is distinct. Nevertheless, the more compact nature of circVLPs vs. circRNA indicates that packaging indeed occurs. ‘Complete’ circVLP assemblies were observed with continuous negative staining – data also indicate that partial assemblies were formed where nucleation did not reach completion (Fig. 4D). This may be due to sub-optimal circRNA: CP mass ratio reactions, strength of the protein-protein and bond interactions of PVX CP during protein-RNA binding, or angular steric hindrance due to the presence of the SL1 RNA structure.

To assess the ability of EGFP-SL1 circVLPs to deliver circRNA to HEK 293T cells, flow cytometry and confocal microscopy were used to measure lipofected cells expressing EGFP over the course of 96 h (4 days) (Fig. 4F-G). Due to the unavailability of a reliable nuclease for circRNA degradation, circVLPs were double sucrose-cushion purified before transfections. circVLPs demonstrated comparable transfection efficiencies as compared to the free EGFP-SL1 circRNA. Flow cytometry results were consistent with confocal microscopy results, showcasing average delivery efficiencies of the 125 µg circVLP were non-inferior to the circRNA positive control until the day 3 timepoint (Fig. 4F). The lower 50 µg circVLP condition also displayed relatively good levels of expression as compared to the positive and negative controls, demonstrating that the EGFP-SL1 mRNA cargo is intact and capable of translation even at lower dosages (Fig. 4F).

## Discussion

We demonstrate here that the plant virus PVX and its CPs provide a versatile nanotechnology platform enabling packaging and delivery of RNA therapeutics. PVX VLPs can package diverse RNAs of various lengths (ranging up to 6.4 kb), sequence, and shape (linear mRNA vs. circRNA) – and the nucleoprotein assemblies can be formed from native or mixtures of native and His-tagged proteins thus providing a means for functionalization (e.g. in future work, targeting ligands could be introduced through complexation via Ni-NTA, similar to a previously reported study using PVX modified with TRAIL (a therapeutic protein engaging death receptors on tumor cells)^31^.

Size-control experiments indicate that more optimization is needed. In theory the RNA should act as a ruler defining the length of the nucleoprotein assembly – however in our studies only a trend could be observed with longer templates leading to VLPs larger in length. Nevertheless, precise size-control was not achieved, and this may be explained by the contamination of assembly reactions larger RNA fragments (residual gRNA, or end-to-end or overlapping alignments of smaller genomic fragment mRNAs forming multiplets) – we also cannot rule out the overextension of the CP nucleation mechanism leading to empty ends. More research is needed to optimize the assembly conditions; defining length control is expected to tailor cell uptake and pharmacokinetic properties. The optimization of recombinant PVX CP production or wildtype PVX CP purification (free of residual gRNA templates) may enable more consistent batching of VLPs encapsulating different RNA templates, leading to more tunable particle properties. Future in vivo and cell uptake studies will allow for a mechanistic understanding of the relationship of size and shape (rod-shaped vs. filamentous) in regard to the cell targeting properties and target gene expression vs. immunomodulatory effects of PVX VLPs.

Concurrently, to enable engineering and future integration with cell targeting or additional therapeutic capabilities, we achieved mixed assembly VLPs incorporating recombinant His-tags; the mixed assemblies retained their structural integrity when a minimum ratio of native CP: rCP-His of 3:7 was mixed. We theorize that this is due to a cooperative binding of CP subunits that involves a minimum number of wildtype CPs (or untagged CPs) to contribute to the protein-protein and protein-RNA interactions that initiate nucleation. Here the His-tag was used to label the VLPs with gold, but the concepts could be expanded to incorporate therapeutic proteins and targeting ligands. Opportunity also exists to produce recombinant CPs with target functionality directly incorporatedas N-terminal fusion. Optimization of the assembly conditions likely would be required considering the His-Tag is a rather small modifier (1.8 kDa). In combination with the inclusion of synthetic mRNAs, these mixed assembly VLPs could also be utilized as powerful RNA therapeutics technologies with dual effector actions, such as modifying the surface CPs with tumor-targeting ligands and encapsulating mRNAs encoding a therapeutic cytokine, where payloads and surface properties could be used to achieve a combined therapeutic aim.

We have also shown the ability of PVX VLPs to encapsulate synthetic foreign mRNAs of different classes – specifically linear and circRNAs – highlighting the platform’s adaptable nature. These studies feature the versatility of PVX as a flexible nucleoprotein assembly technology – so flexible indeed that the assembly of halo-shaped PVX packaging circRNA was achieved. Cell transfection using PVX VLPs packaging linear mRNA or circRNA was accomplished and were non-inferior to control RNA conditions. Additionally, the high dosage of VLPs per cell in culture corresponds to a lower cytotoxicity, higher biocompatibility, and a promising safety profile.

We note some limitations of this work: a major challenge in the mRNA delivery field is to increase shelf-life and stability of the carrier system to avoid cold chain requirements. While plant VLPs are generally thought to be highly stable carriers evolved to persist in various environmental conditions, stability of the VLP system must be assessed in future studies. Finally, once the system is more optimized, head-to-head comparisons with existing technologies such as LNPs and mammalian viral vector systems must be completed to demonstrate the competitiveness of the proposed system.

## Supplementary Information

Below is the link to the electronic supplementary material.

Supplementary Material 1

## Data Availability

Data available from the corresponding author (Nicole F. Steinmetz, Ph.D., [nsteinmetz@ucsd.edu] (mailto: nsteinmetz@ucsd.edu)) upon reasonable request.
