# Supplementary material for: Engineering filamentous potato virus X as a platform nanotechnology for nucleic acid gene delivery
Source: Sci Rep. 2025 Dec 12;15:43714. doi: 10.1038/s41598-025-27488-7 (PMC12700909; doi:10.1038/s41598-025-27488-7)
Supplement: Supplementary file 1 — Supplementary Material 1 [file 41598_2025_27488_MOESM1_ESM.pdf]

**Supplementary Information for**

# Engineering Filamentous Potato Virus X as a Platform Nanotechnology for Nucleic Acid Gene Delivery

**Authors:**
Bryan Duoto[1,3,4,5], Michael Tong[2], Krister J. Barkovich[3,4,5,6], Juliane Schuphan[7], Prashant Mali[2], and Nicole F. Steinmetz[1,3,4,5,6,8,9,10]

[1] Aiiso Yufeng Li Family Department of Chemical and Nano Engineering, University of California San Diego, 9500 Gilman Dr., La Jolla, California 92093, United States

[2] Shu Chien-Gene Lay Department of Bioengineering, University of California, San Diego, La Jolla, California 92093, United States.

[3] Center for Nano-ImmunoEngineering, University of California, San Diego, La Jolla, California 92093, United States.

[4] Moores Cancer Center, University of California, San Diego, La Jolla, California 92093, United States.

[5] Shu and K. C. Chien and Peter Farrell Collaboratory, University of California, San Diego, La Jolla, California 92093, United States.

[6] Department of Radiology, University of California, San Diego, La Jolla, California 92093, United States.

[7] Institut für Molekulare Biotechnologie, RWTH Aachen University, Worringer Weg 1, 52074 Aachen, Germany

[8] Department of Bioengineering, University of California, San Diego, La Jolla, California 92093, United States.

[9] Institute for Materials Discovery and Design, University of California, San Diego, La Jolla, California 92093, United States.

[10] Center for Engineering in Cancer, Institute for Engineering in Medicine, University of California, San Diego, La Jolla, California 92093, United States.

[#] Corresponding author: nsteinmetz@ucsd.edu

## Supplemental Figures

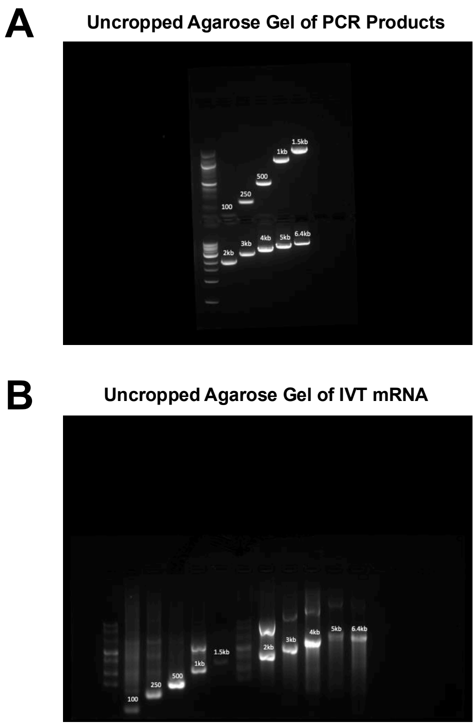


**Figure S1.** Uncropped gel images as shown in Figure 1B. (A) Uncropped 1.2% (w/v) agarose gel in TBE buffer of PCR products used for generating mRNA transcripts. (B) Uncropped 1.2% (w/v) agarose gel in TBE buffer of mRNA transcripts derived from PCR products via *in vitro* transcription.

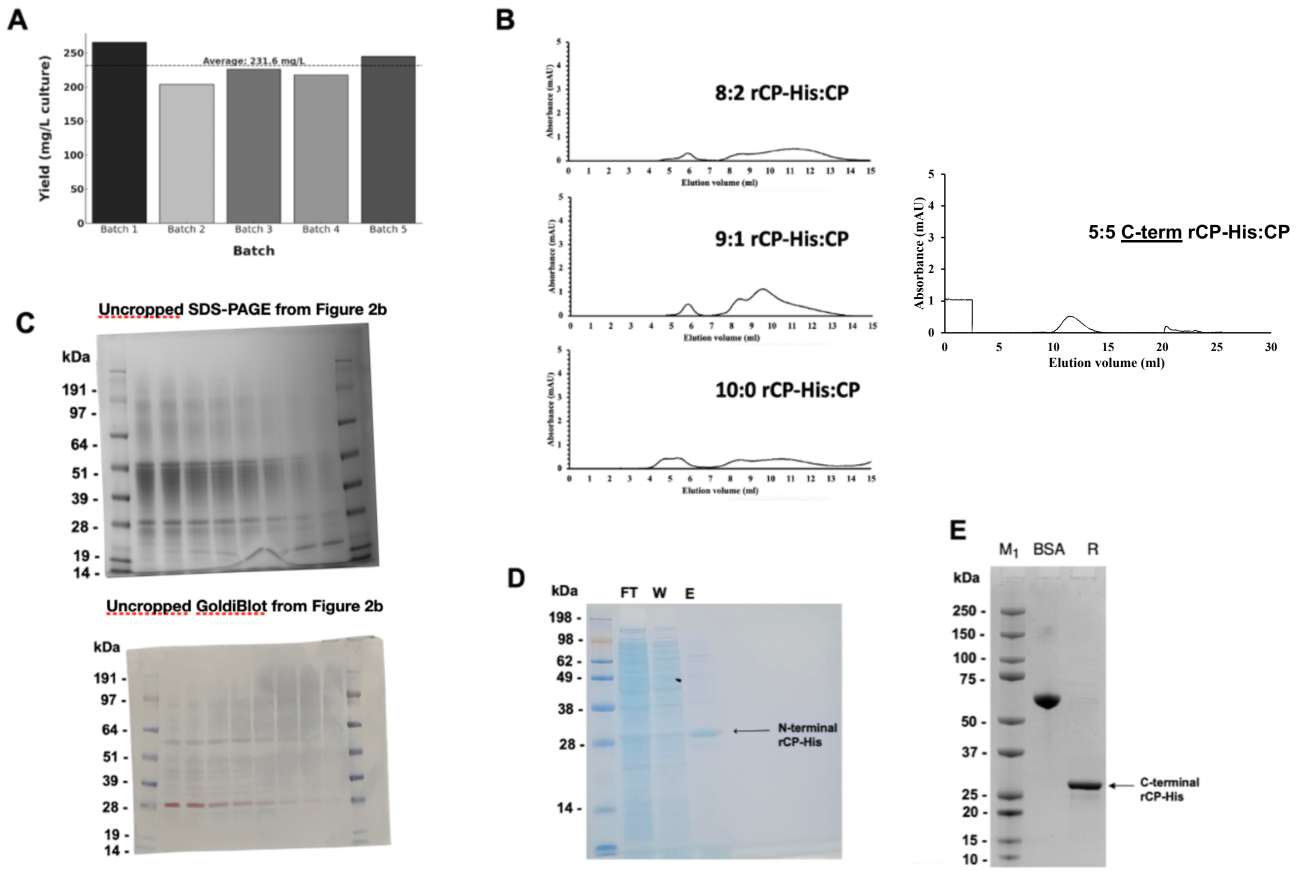


**Figure S2.** Supplemental information on mixed assembly VLPs. (A) Yields of N-terminal 9x His-tagged CPs after purification from (DE3) BL21 cells. (B) Size exclusion chromatography (SEC) of the different mixed assembly VLP conditions after assembly and purification for ratio combinations that did not reliably produce VLPs; an example of mixed assembly using rCP-His with C-terminal His tag and native CP is also included to showcase lack of assembly – the same condition yielded VLPs when rCP-His with N-terminal His-tag was used. (C) Uncropped Goldiblot Western gel with ladder displaying VLPs at a characteristic molecular weight of ~28kDa; see also **Figure 2B**. (D) Purification of rCP-His from bacterial pellets including lanes for the flow-through (FT), wash (W), and elution (E) samples in a 4-12% Bis-Tris SDS-PAGE gel. (E) Purified C-terminal rCP-His (R) was also derived from bacterial cultures and exhibited >90% purity as compared to BSA, however C-terminal rCP-His did not lead to successful VLP formation in assembly conditions tested (see panel 2B).

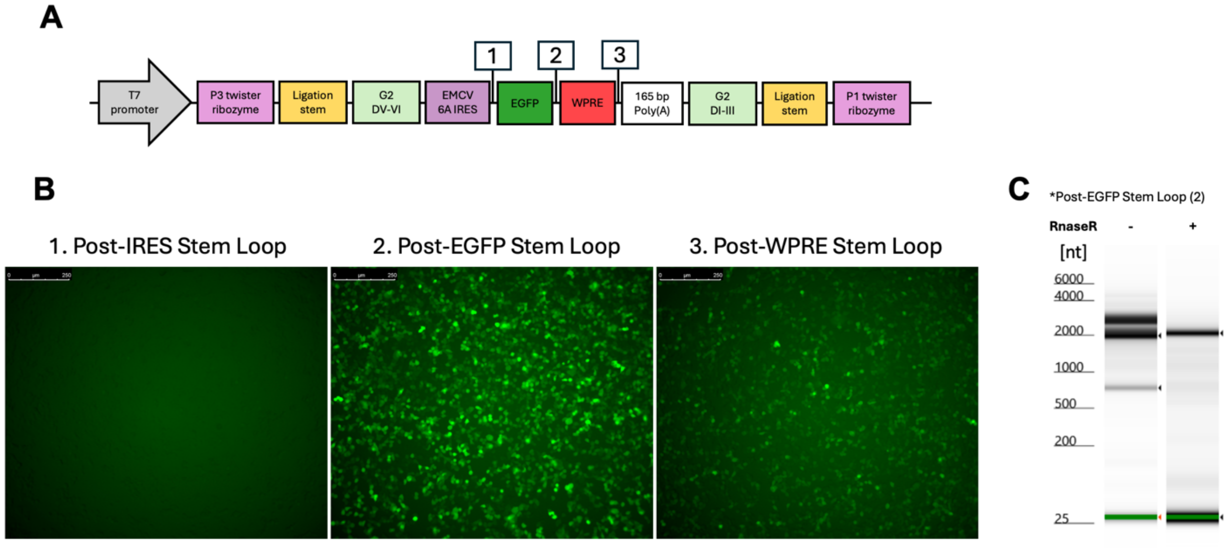


**Figure S3.** Supplemental information on circVLPs and the design rationale of circRNA. (A) Three different locations were chosen for insertion of the SL-1 OAS site to study the effects of the RNA stem loop on circularization and expression of the reporter gene. (B) After transfection of circRNAs into HEK 293T cells using Lipofectamine 2,000, delivery efficiencies were observed across the three locations with the post-EGFP SL1 working the best. (C) The circRNAs from the post-EGFP SL1 condition were shown to successfully circularize and were purified away from linear pre-circRNA transcripts and free introns.

## Supplemental Tables

### Supplementary Table S1: gBlocks

| | |
|---|---|
| SL1 cloned into pCMV-T7-EGFP | TTTAGTGAACCGTCAGATCCGCTAGAGATCCGCGGCCGCTAAT<br>ACGACTCACTATAGGGAGAGCCTTGTTACACACCCGCTTGAAA<br>AAGCAAGTCTGACAAAAGGCCAAAGTGCGCGAGGGCCACCATG<br>GTGAGCAAGGGCGAGGAGCTGTTCACCGGGGTGGTGCC |
| SL1 cloned into Circ-ECMV-GFP | GTGCTGGTTATTGTGCTGTCTCATCATTTTGGCAAAGCGGCCG<br>CGGCCAGTGAATTGTAATACGACTCACTATAGGGGTTAGAGCC<br>TTGTTACACACCCGCTTGAAAAAGCAAGTCTGACAAAAGGCCA<br>AAGTGCGCGAGGGCCACCGGTAACAAAAAAAAAAAAAAAAAAA<br>AAAAATCACCGACCGATCTATCTGAACCGGTGGGAATTCTAGA<br>G |
| PVX Genome Segment 1 cloned into pCMV-T7<br>1 nt-2131 nt of the PVX Genome | TAATACGACTCACTATAagagccGAAAACTAAACCATACACCA<br>CCAACACAACCAAACCCACCACGCCCAATTGTTACACACCCGC<br>TTGGAAAAGCAAGTCTAACAAATGGCCAAAGTGCGCGAGGTTT<br>ACCAATCCTTTACAGACTCCACCACAAAAACTCTCATCCAAGA<br>TGAGGCTTATAGAAATATTCGCCCCATCATGGAAAAACACAAA<br>CTAGCTAACCCTTACGCTCAAACGGTTGAAGCGGCTAATGATC<br>TAGAGGGGTTCGGCATAGCCACCAATCCCTATAGCATTGAATT<br>GCATACACATGCAGCCGCTAAGACCATAGAGAATAAACTTCTA<br>GAGGTGCTTGGTTCCATCCTACCACAAGAACCTGTTACATTTA<br>TGTTCCTTAAACCCAGGAAGCTAAACTACATGAGAAGAAACCC<br>GCGGATCAAGGACATTTTCCACAATGTTGCCATTGAACCGAGA<br>GACGTAGCAAGGTACCCCAAGGAAACAATAATTGACAAACTCA<br>CAGAGATCACAACGGAAACAGCATACATTAGTGATACTCTGCA<br>CTTCTTGGATCCGAGCTACATAGTGGAGACATTCCAAAACTGC<br>CCAAAACTGCAAACATTGTATGCGACCTTAGTTCTCCCCGTTG<br>AGGCAGCCTTCAAAATGGAAAGCACTCACCCGAACATATACAG<br>CCTCAAATACTTCGGAGATGGTTTCCAGTATATACCAGGCAAC<br>CATGGTGGTGGGGCATACCATCATGAATTCGCTCATTTACAAT<br>GGCTCAAAGTGGGAAAGATCAAGTGGAGGGACCCCAAGGATAG<br>CTTTCTCGGACATCTCAATTACACGACTGAGCAGGTTGAGATG<br>CACACAGTGACAGTACAGTTGCAGGAATCGTTTGCGGCAAACC<br>ACTTGTACTGCATTAGGAGAGGAGACTTGCTCACACCGGAGGT<br>GCGTACTTTCGGCCAACCTGACAGGTATGTGATTCCACCACAG<br>ATCTTTCTCCCAAAAGTTCACAACTGCAAGAAGCCGATTCTTA<br>AGAAAACTATGATGCAGCTCTTCTTGTATGTTAGGACAGTCAA<br>GGTCGCAAAAAATTGTGACATCTTTGCCAAAGTCAGACAATTA<br>ATTAAATCATCTGACTTAGACAAATATTCTGCTGTGGAACTGG<br>TTTACTTAGTAAGCTATATGGAGTTCCTTGCCGATTTACAAGC<br>TACCACCTGCTTCTCAGACACACTCTCTGGTGGCTTGCTAACA<br>AAGACCCTTGCACCGGTGAGGGCTTGGATACAAGAAAAGAAGA<br>TGCAGCTGTTTGGTCTTGAGGACTACGCGAAGTTAGTCAAAGC<br>AGTTGATTTCCACCCGGTGGATTTTTCTTTCAAAGTGGAAACT<br>TGGGACTTCAGATTCCACCCCTTGCAAGCGTGGAAAGCCTTCC<br>GACCAAGGGAAGTGTCGGATGTAGAGGAAATGGAAAGTTTGTT<br>CTCAGATGGGGACCTGCTTGATTGCTTCACAAGAATGCCAGCT<br>TATGCAGTAAACGCAGAGGAAGATTTAGCTACAATCAGGAAAA<br>CGCCCGAGATGGATGTCGGTCAAGAAGTTAAAGAGCCTGCAGG<br>AGACAGAAATCAATACTCAAACCCTGCAGAAACTTTCCTCAAC<br>AAGCTCCACAGGAAACACAGTAGGGAGGTGAAACACCAGGCCG<br>TAAAGAAAGCTAAACGCCTAGCTGAAATCCAGGAGTCCATGAG |

| | |
|---|---|
| | AGCTGAAGGTGAGGCCGAACCAAATGAGACGAGCGGGGGCATG<br>GGGGCAATACCCAGCAACGCCGAACTTCCCGGCACGAGTGATG<br>CCAGACAAGAACTCACACTCCCAACCACTAAACCTGTTCCTGC<br>AAGGTGGGAAGATGCTTCATTCACAGATTCTAGTGTGGAAGAG<br>GAGCAGGTAAGACTCCTTGGAGAAGAAGCAGTGAAAACAGCGA<br>CGCAGCAAGTCATCGAAGGACTCCCTTGGAAACACTGGATTCC<br>TCAACTAAATGCTGTTGGATTCAAGGCGCTGTTAATCCAGAGG<br>GATAGGAGTGGAACGATGATCATGCCCATCACAGAAATGGTCT<br>CCGGGTTGGAAAAAGAGGACTTCCCGGAAGGAACTCCAAAAGA<br>GTTGGCACGAGAATTACTCGTTATGAACAGAAGCCCTGCCACC<br>ATCCCTTTGGACCTGCTTAGAGC |
| PVX Genome Segment 2 cloned into pCMV-T7<br>2131 nt-3813 nt of the PVX Genome | TTGGCACGAGAATTACTCGTTATGAACAGAAGCCCTGCCACCA<br>TCCCTTTGGACCTGCTTAGAGCCAGAGACTACGGCAGTGATGT<br>AAAGAACAAGAGAATTGGTGCCATCACAAAGACACAGGCAACG<br>AGTTGGGGCGAGTACTTAACAGGAAAGATAGAAAGCCTGACTG<br>AGAGGAAAGTTGCGACTTGTGTCATTCATGGAGCTGGAGGCTC<br>TGGGAAAAGTCATGCCATCCAGAAGGCACTGAGAGAAATTGGC<br>AAGGGCTCGGACATCACTGTAGTCCTGCCGACCAATGAACTGC<br>GGCTAGATTGGAGTAAGAAGGTGCCTAACACTGAACCCTATAT<br>GTTCAAGACCTACGAAAAGGCGTTAATTGGGGGAACAGGCAGC<br>ATAGTCATCTTTGACGATTACTCAAAACTTCCTCCCGGTTACA<br>TAGAAGCCTTAGTCTGTTTCTACTCCAAAATCAAGCTAATCAT<br>TCTAACAGGAGATAGCAGACAGAGCGTCTACCATGAAACTGCT<br>GAGGACGCCTCCATCAGGCATTTGGGGCCAGCGACAGAGTACT<br>TCTCAAAATACTGCCGATACTATCTCAATGCTACACACCGCAA<br>CAAGAAAGACCTTGCGAACATGCTTGGTGTCTACAGTGAGAGA<br>ACGGGAGTCACTGAAATCAGCATGAGCGCCGAGTTTTTAGAAG<br>GAATCCCAACTTTAGTACCCTCGGATGAGAAGAGAAAGCTGTA<br>CATGGGCACCGGGAGGAATGACACATTCACATACGCTGGATGC<br>CAGGGGCTAACTAAGCCGAAAGTACAAATAGTGTTGGACCACA<br>ACACCCAAGTGTGTAGTGCGAATGTGATGTACACGGCACTTTC<br>TAGAGCCACCGATAGGATTCACTTCGTGAACACAAGTGCAAAC<br>TCCTCGGCCTTCTGGGAAAAGTTGGACAGCACCCCTTACCTCA<br>AGACTTTCCTATCAGTGGTGAGAGAACAAGCACTCAGGGAGTA<br>TGAGCCGGCAGAGGCAGAGCCAATTCAAGAGCCTGAGCCCCAG<br>ACACACATGTGTGTCGAGAATGAGGAGTCCGTGCTAGAAGAGT<br>ACAAAGAGGAACTCTTGGAAAAGTTTGACAGAGAGATACACTC<br>TGAATCCCATGGTCATTCAAACTGTGTCCAAACTGAAGACACA<br>ACCATTCAGTTGTTTTCGCATCAACAAGCAAAAGATGAGACCC<br>TCCTCTGGGCGACCATAGATGCGCGGCTCAAGACTAGCAATCA<br>AGAGGCAAACTTCCGAGAATTCCTGAGCAAGAAGGACATTGGG<br>GACGTTCTGTTTTTAAACTACCAAAAAGCTATGGGTTTGCCCA<br>AAGAGCGTATTCCTTTTTCCCAAGAGGTCTGGGAAGCTTGTGC<br>CCACGAAGTACAAAGCAAGTACCTCAGTAAGTCAAAGTGCAAC<br>TTGATCAATGGGACTGTGAGACAGAGCCCAGACTTCGATGAAA<br>ACAAGATTATGGTATTCCTCAAGTCGCAGTGGGTCACAAAGGT<br>GGAAAAACTAGGTCTACCCAAGATTAAGCCAGGTCAAACCATA<br>GCAGCCTTTTACCAGCAGACTGTGATGCTTTTTGGAACTATGG<br>CTAGGTACATGCGATGGTTCAGACAGGCTTTCCAGCCAAAAGA<br>AGTCTTCATAAACTGTGAGACTACGCCAGAAGACATGTCTGTA<br>TGGGCCTTGAACAACTGGAATTTCAGCAGACCTAGCTTAGCTA<br>ATGACTAC |

| | |
|---|---|
| PVX Genome Segment 3 cloned into pCMV-T7<br>3813 nt-5311 nt of the PVX Genome | TGTATGGGCCTTGAACAACTGGAATTTCAGCAGACCTAGCTTA<br>GCTAATGACTACACAGCTTTCGACCAGTCTCAGGATGGAGCTA<br>TGCTGCAATTTGAGGTGCTCAAAGCCAAGCACCACTGCATACC<br>AGAGGAAATCATCCAAGCATACATAGACATTAAGACCAATGCA<br>CAGATTTTCCTAGGCACATTATCGATTATGCGCCTGACTGGTG<br>AGGGTCCCACTTTTGATGCAAACACTGAGTGCAACATAGCTTA<br>CACCCACACAAAGTTTGACATCCCAGCCGGAACTGCTCAAGTT<br>TATGCAGGAGACGACTCCGCACTGGATTGCGTTCCAGAAGTGA<br>AGCATAGTTTCCACAGGCTTGAAGACAAATTACTCCTCAAGTC<br>AAAGCCTGTAATCACGCAGCAAAAGAAAGGCAGTTGGCCTGAG<br>TTTTGTGGTTGGCTGATCACACCAAAAGGGGTAATGAAAGACC<br>CAATTAAGCTCCATGTTAGCTTAAAATTGGCCGAAGCTAAGGG<br>TGAACTCAAGAAATGTCAAGATTCCTATGAAATTGATCTGAGT<br>TATGCCTATGACCACAAGGACTCTCTGCATGACTTGTTCGATG<br>AGAAACAGTGTCAGGCACATACACTCACTTGCAGGACACTAAT<br>CAAGTCAGGGAGAGGCACTGTCTCACTTCCCCGCCTCAGAAAC<br>TTTCTTTAACCGTTAATTTACCTTATAGATTTGAATAAGATGG<br>ATATTCTCATCAGTAGTTTGAAAAGTTTAGGTTATTCTAGGAC<br>TTCTAAATCTTTAGATTCAGGACCTTTGGTAGTACATGCAGTA<br>GCCGGAGCAGGTAAGTCCACAGCCCTAAGGAAGTTGATCCTCA<br>GACACCCAACATTCACCGTGCATACACTCGGTGTCCCTGACAA<br>GGTGAGTATCAGAACTAGAGGCATACAGAAGCCAGGACCTATT<br>CCTGAGGGCAATTTCGCAATCCTCGATGAGTATACTTTGGACA<br>ACACCACAAGGAACTCATACCAGGCACTTTTTGCTGACCCTTA<br>TCAGGCACCTGAGTTTAGCCTAGAGCCCCACTTCTACTTGGAA<br>ACATCATTTCGAGTTCCGAGGAAAGTGGCAGATTTGATAGCTG<br>GCTGTGGCTTCGATTTCGAGACTAACTCACAGGAAGAAGGGCA<br>TTTAGAGATCACTGGCATATTCAAAGGGCCCCTACTTGGAAAG<br>GTGATAGCCATTGATGAGGAGTCTGAGACAACACTGTCCAGGC<br>ATGGTGTTGAGTTTGTTAAGCCCTGCCAAGTGACTGGACTTGA<br>GTTGAAAGTAGTCACTATTGTGTCTGCCGCACCAATAGAGGAA<br>ATTGGCCAGTCCACAGCTTTCTACAACGCTATCACCAGGTCAA<br>AGGGATTGACATATGTCCGCGCAGGGACATAGACTGACCGCTC<br>CGGTCAATTCTGAAAAAGTGTACATAGTATTAGGTCTATCATT<br>TGCTTTAGTTTCAATTACTTTCCTGCTTTCTAGAAATAGTTTG<br>CCCCACGTCGGTGACAACATTCACAGCTTGCCACACGGAGGAG<br>CTTAC |
| PVX Genome Segment 4 cloned into pCMV-T7<br>5311 nt-6435 nt of the PVX Genome | GACCGCTCCGGTCAATTCTGAAAAAGTGTACATAGTATTAGGT<br>CTATCATTTGCTTTAGTTTCAATTACTTTCCTGCTTTCTAGAA<br>ATAGTTTGCCCCACGTCGGTGACAACATTCACAGCTTGCCACA<br>CGGAGGAGCTTACAGAGACGGCACCAAAGCAATCTTGTACAAC<br>TCCCCAAATCTAGGGTCACGAGTGAGTCTACACAACGGAAAGA<br>ACGCAGCATTTGCTGCCGTTTTGCTACTGACTTTGCTGATCTA<br>TGGAAGTAAATACATATCTCAACGCAATCATACTTGTGCTTGT<br>GGTAACAATCATAGCAGTCATTAGTACTTCCTTAGTGAGGACT<br>GAACCTTGTGTCATCAAGATTACTGGGGAATCAATCACAGTGT<br>TGGCTTGCAAATTAGATGCAGAAACTATAAAAGCCATTGCCGA<br>TCTCAAGCCACTCTCCGTTGAACGGTTAAGTTTCCATTGATAC<br>TCGAAAGATGTCAGCACCAGCTAGCACAACACAGGCCACAGGG<br>TCAACTACCTCAACTACCACGAAAACTGCAGGCGCAACTCCTG<br>CCACAGCTTCAGGCCTGTTCACCATCCCGGATGGGGATTTCTT<br>TAGTACAGCTCGTGCCATAGTAGCCAGCAATGCTGTCGCAACA<br>AATGAGGACCTCAGCAAGATTGAGGCTATTTGGAAGGACATGA<br>AGGTGCCCACAGACACTATGGCACAGGCTGCTTGGGACTTAGT<br>CAGACACTGTGCTGATGTGGGATCATCTGCTCAAACAGAAATG<br>ATAGATACAGGTCCTTATTCCAACGGCATCAGCAGAGCTAGAC |

| | |
|---|---|
| | TGGCAGCAGCAATCAAAGAGGTGTGCACACTTAGGCAATTTTG<br>CATGAAGTATGCTCCAGTGGTATGGAACTGGATGTTAACTAAC<br>AACAGTCCACCTGCTAACTGGCAAGCACAAGGTTTCAAGCCTG<br>AGCACAAATTCGCTGCATTCGACTTCTTCAATGGAGTCACCAA<br>CCCAGCTGCCATCATGCCCAAAGAGGGGCTCATCCGGCCACCG<br>TCTGAAGCTGAAATGAATGCTGCCCAAACTGCTGCTTTTGTGA<br>AGATTACAAAGGCCAGGGCACAATCCAACGACTTTGCCAGCCT<br>AGATGCAGCTGTCACTCGAGGTCGTATCACTGGAACAACAACC<br>GCTGAGGCTGTTGTCACTCTACCACCACCATAACTACGTCTAC<br>ATAACCGACGCCTACCCCAGTTTCATAGTATTTTCTGGTTTGA<br>TTGTATGAATAATATAAATTCTAGAGCTGGAGCCTCGGTAGCC<br>GTTCCTCCTGCCCGCTGGGCCTCCCAACGGGCCCTCCTCCCCT<br>CCTTGCACCGGCCCTTCCTGGTCTTTGCCGCGGCTGTGCCTTC<br>TAGTTGCCAGCCATCTGTTGTTTGCCCCTCCCCCGTGCC |
| PVX CP into pHGWA-9xHis | AGATCCAGATCTCGATCCCGCGAAATTAATACGACTCACTATA<br>GGGGAATTGTGAGCGGATAACAATTCCCCTCTAGAAATAATTT<br>TGTTTAACTTTAAGAAGGAGATATACCATGGGCAGCAGCCATC<br>ATCATCATCATCATCATCATCACGGTGAGAATCTTTATTTTCA<br>GGGCATGTCAGCACCAGCTAGCACAACACAGACCATAGGGTCA<br>ACTACCTCAACTACCACAAAAACTGCAGGCGCAACTCCTGCCA<br>CAGCTTCAGGACTGTTCACCATCCCGGATGGGGATTTCTTTAA<br>CACAGCCCGTGCCATAGTAGCCAGCAATGCCGTTGCAACGAAT<br>GAGGACCTCAGAAAGATTGAGGCTATTTGGAAAGACATGAAGG<br>TACCCACAGACACTATGGCACAGGCTGCTTGGGACTTGGTCAG<br>ACACTGTGCTGATGTGGGCTCATCTGCCCAAACAGAGATGATA<br>GATACAGGTCCTTATTCCAATGGCATCAGCAGAGCTAGACTGG<br>CAGCAGCGATTAAAGAGGTGTGCACACTTAGACAATTCTGCAT<br>GAAGTATGCCCCAGTGGTATGGAACTGGATGTTGACTAACAAC<br>AGTCCACCTGCTAACTGGCAAGCACAAGGTTTCAAGCCTGAGC<br>ACAAATTCGCTGCATTCGACTTCTTCAACGGAGTCACCAACCC<br>AGCTGCCATCATGCCCAAAGAGGGACTCATCCGGCCACCATCT<br>GAAGCAGAAATGAATGCTGCCCAAACTGCTGCCTTTGTGAAAA<br>TTACGAAGGCCAGGGCACAATCCAACGACTTTGCCAGCCTAGA<br>TGCGGCTGTCACTCGAGGTCGTATCACTGGAACAACAACCGCT<br>GAGGCTGTTGTCACTCTACCACCACCATAAAAGGGTGGGCGCG<br>CCGACCCAGCTTTCTTGTACAAAGTGGTGATGTACCTCGAGCA<br>CCACCACCACCACCAC |

## Supplementary Table S2: Plasmid maps and sequences

## pCMV-T7-SL1-EGFP

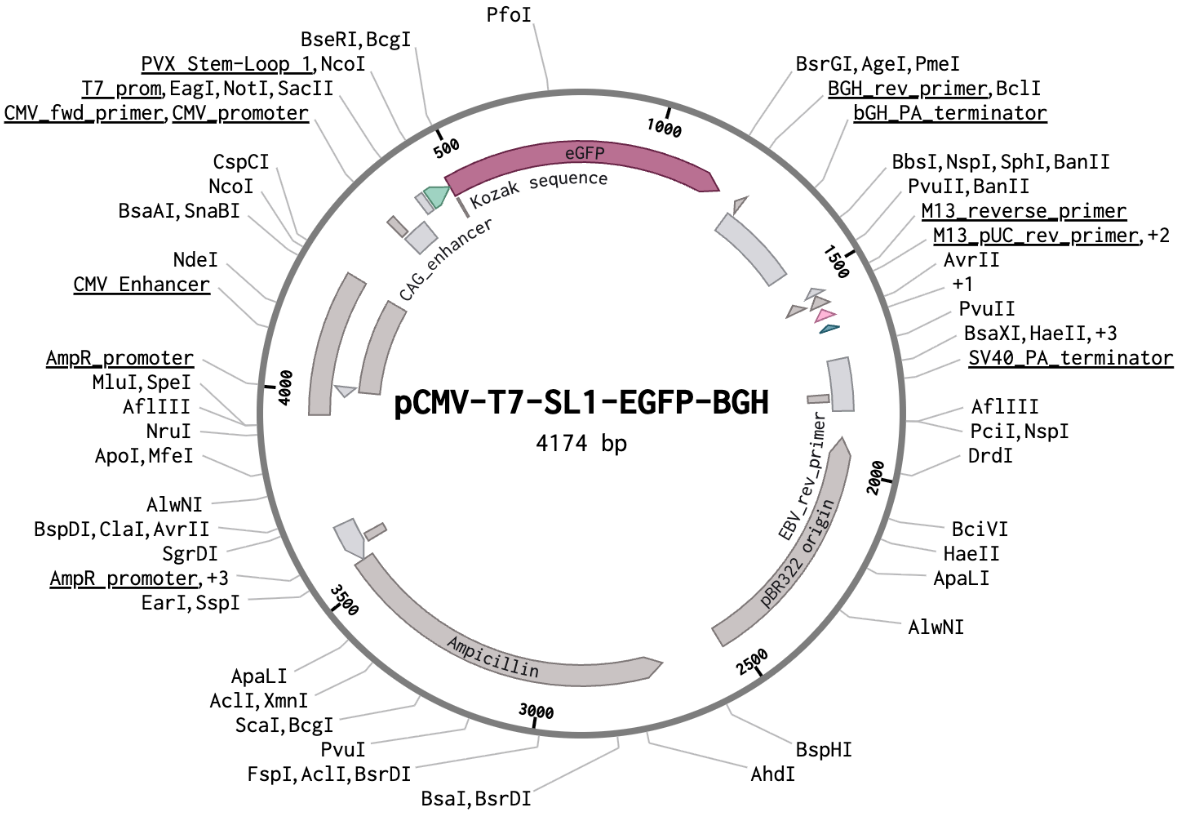


Sequence:

**ATATGCCAAGTACGCCCCCTATTGACGTCAATGACGGTAAATGGCCCGCCTGGCATTATGCCCAGTACATGACCTTA
TGGGACTTTCCTACTTGGCAGTACATCTACGTATTAGTCATCGCTATTACCATGGTGATGCGGTTTTGGCAGTACAT
CAATGGGCGTGGATAGCGGTTTGACTCACGGGGATTTCCAAGTCTCCACCCCATTGACGTCAATGGGAGTTTGTTTT
GGCACCAAAATCAACGGGACTTTCCAAAATGTCGTAACAACTCCGCCCCATTGACGCAAATGGGCGGTAGGCGTGTA
CGGTGGGAGGTCTATATAAGCAGAGCTGGTTTAGTGAACCGTCAGATCCGCTAGAGATCCGCGGCCGCTAATACGAC
TCACTATAGGGAGAGCCTTGTTACACACCCGCTTGAAAAAGCAAGTCTGACAAAAGGCCAAAGTGCGCGAGGGCCAC
CATGGTGAGCAAGGGCGAGGAGCTGTTCACCGGGGTGGTGCCCATCCTGGTCGAGCTGGACGGCGACGTAAACGGCC
ACAAGTTCAGCGTGTCCGGCGAGGGCGAGGGCGATGCCACCTACGGCAAGCTGACCCTGAAGTTCATCTGCACCACC
GGCAAGCTGCCCGTGCCCTGGCCCACCCTCGTGACCACCCTGACCTACGGCGTGCAGTGCTTCAGCCGCTACCCCGA
CCACATGAAGCAGCACGACTTCTTCAAGTCCGCCATGCCCGAAGGCTACGTCCAGGAGCGCACCATCTTCTTCAAGG
ACGACGGCAACTACAAGACCCGCGCCGAGGTGAAGTTCGAGGGCGACACCCTGGTGAACCGCATCGAGCTGAAGGGC
ATCGACTTCAAGGAGGACGGCAACATCCTGGGGCACAAGCTGGAGTACAACTACAACAGCCACAACGTCTATATCAT
GGCCGACAAGCAGAAGAACGGCATCAAGGTGAACTTCAAGATCCGCCACAACATCGAGGACGGCAGCGTGCAGCTCG
CCGACCACTACCAGCAGAACACCCCCATCGGCGACGGCCCCGTGCTGCTGCCCGACAACCACTACCTGAGCACCCAG
TCCGCCCTGAGCAAAGACCCCAACGAGAAGCGCGATCACATGGTCCTGCTGGAGTTCGTGACCGCCGCCGGGATCAC
TCTCGGCATGGACGAGCTGTACAAGTGACCGGTCATCATCACCATCACCATTGAGTTTAAACCCGCTGATCAGCCTC
GACTGTGCCTTCTAGTTGCCAGCCATCTGTTGTTTGCCCCTCCCCCGTGCCTTCCTTGACCCTGGAAGGTGCCACTC
CCACTGTCCTTTCCTAATAAAATGAGGAAATTGCATCGCATTGTCTGAGTAGGTGTCATTCTATTCTGGGGGGTGGG**

GTGGGGCAGGACAGCAAGGGGGAGGATTGGGAAGACAATAGCAGGCATGCTGGGGATGCGGTGGGCTCTATGGCTTC
TGAGGCGGAAAGAACCAGCTGGGGCTCGATACCGTCGACCTCTAGCTAGAGCTTGGCGTAATCATGGTCATAGCTGT
TTCCTGTGTGAAATTGTTATCCGCTCACAATTCCACACAACATACGAGCCGGAAGCATAAAGTGTAAAGCCTAGGGT
GCCTAATGAGTGAGCTAACTCACATTAATTGCGTTGCGCTCACTGCCCGCTTTCCAGTCGGGAAACCTGTCGTGCCA
GCTGCATTAATGAATCGGCCAACGCGCGGGGAGAGGCGGTTTGCGTATTGGGCGCTCTTCCGCTTCCTCGCTCACTG
ACTCGCTGCGCTCGGTCGTTCGGCTGCGGCGAGCGGTATCAGCTCACTCAAAGGCGGTAATACGGTTATCCACAGAA
TCAGGGGATAACGCAGGAAAGAACATGTGAGCAAAAGGCCAGCAAAAGGCCAGGAACCGTAAAAAGGCCGCGTTGCT
GGCGTTTTTCCATAGGCTCCGCCCCCCTGACGAGCATCACAAAAATCGACGCTCAAGTCAGAGGTGGCGAAACCCGA
CAGGACTATAAAGATACCAGGCGTTTCCCCCTGGAAGCTCCCTCGTGCGCTCTCCTGTTCCGACCCTGCCGCTTACC
GGATACCTGTCCGCCTTTCTCCCTTCGGGAAGCGTGGCGCTTTCTCAATGCTCACGCTGTAGGTATCTCAGTTCGGT
GTAGGTCGTTCGCTCCAAGCTGGGCTGTGTGCACGAACCCCCCGTTCAGCCCGACCGCTGCGCCTTATCCGGTAACT
ATCGTCTTGAGTCCAACCCGGTAAGACACGACTTATCGCCACTGGCAGCAGCCACTGGTAACAGGATTAGCAGAGCG
AGGTATGTAGGCGGTGCTACAGAGTTCTTGAAGTGGTGGCCTAACTACGGCTACACTAGAAGGACAGTATTTGGTAT
CTGCGCTCTGCTGAAGCCAGTTACCTTCGGAAAAAGAGTTGGTAGCTCTTGATCCGGCAAACAAACCACCGCTGGTA
GCGGTGGTTTTTTTGTTTGCAAGCAGCAGATTACGCGCAGAAAAAAAGGATCTCAAGAAGATCCTTTGATCTTTTCT
ACGGGGTCTGACGCTCAGTGGAACGAAAACTCACGTTAAGGGATTTTGGTCATGAGATTATCAAAAAGGATCTTCAC
CTAGATCCTTTTAAATTAAAAATGAAGTTTTAAATCAATCTAAAGTATATATGAGTAAACTTGGTCTGACAGTTACC
AATGCTTAATCAGTGAGGCACCTATCTCAGCGATCTGTCTATTTCGTTCATCCATAGTTGCCTGACTCCCCGTCGTG
TAGATAACTACGATACGGGAGGGCTTACCATCTGGCCCCAGTGCTGCAATGATACCGCGAGACCCACGCTCACCGGC
TCCAGATTTATCAGCAATAAACCAGCCAGCCGGAAGGGCCGAGCGCAGAAGTGGTCCTGCAACTTTATCCGCCTCCA
TCCAGTCTATTAATTGTTGCCGGGAAGCTAGAGTAAGTAGTTCGCCAGTTAATAGTTTGCGCAACGTTGTTGCCATT
GCTACAGGCATCGTGGTGTCACGCTCGTCGTTTGGTATGGCTTCATTCAGCTCCGGTTCCCAACGATCAAGGCGAGT
TACATGATCCCCCATGTTGTGCAAAAAAGCGGTTAGCTCCTTCGGTCCTCCGATCGTTGTCAGAAGTAAGTTGGCCG
CAGTGTTATCACTCATGGTTATGGCAGCACTGCATAATTCTCTTACTGTCATGCCATCCGTAAGATGCTTTTCTGTG
ACTGGTGAGTACTCAACCAAGTCATTCTGAGAATAGTGTATGCGGCGACCGAGTTGCTCTTGCCCGGCGTCAATACG
GGATAATACCGCGCCACATAGCAGAACTTTAAAAGTGCTCATCATTGGAAAACGTTCTTCGGGGCGAAAACTCTCAA
GGATCTTACCGCTGTTGAGATCCAGTTCGATGTAACCCACTCGTGCACCCAACTGATCTTCAGCATCTTTTACTTTC
ACCAGCGTTTCTGGGTGAGCAAAAACAGGAAGGCAAAATGCCGCAAAAAAGGGAATAAGGGCGACACGGAAATGTTG
AATACTCATACTCTTCCTTTTTCAATATTATTGAAGCATTTATCAGGGTTATTGTCTCATGAGCGGATACATATTTG
AATGTATTTAGAAAAATAAACAAATAGGGGTTCCGCGCACATTTCCCCGAAAAGTGCCACCTGACGTCGACGGATCG
GGAGATCGATCTCCCGATCCCCTAGGGTCGACTCTCAGTACAATCTGCTCTGATGCCGCATAGTTAAGCCAGTATCT
GCTCCCTGCTTGTGTGTTGGAGGTCGCTGAGTAGTGCGCGAGCAAAATTTAAGCTACAACAAGGCAAGGCTTGACCG
ACAATTGCATGAAGAATCTGCTTAGGGTTAGGCGTTTTGCGCTGCTTCGCGATGTACGGGCCAGATATACGCGTTGA
CATTGATTATTGACTAGTTATTAATAGTAATCAATTACGGGGTCATTAGTTCATAGCCCATATATGGAGTTCCGCGT
TACATAACTTACGGTAAATGGCCCGCCTGGCTGACCGCCCAACGACCCCCGCCCATTGACGTCAATAATGACGTATG
TTCCCATAGTAACGCCAATAGGGACTTTCCATTGACGTCAATGGGTGGACTATTTACGGTAAACTGCCCACTTGGCA
GTACATCAAGTGTATC

## Circ-EMCV-GFP-SL1

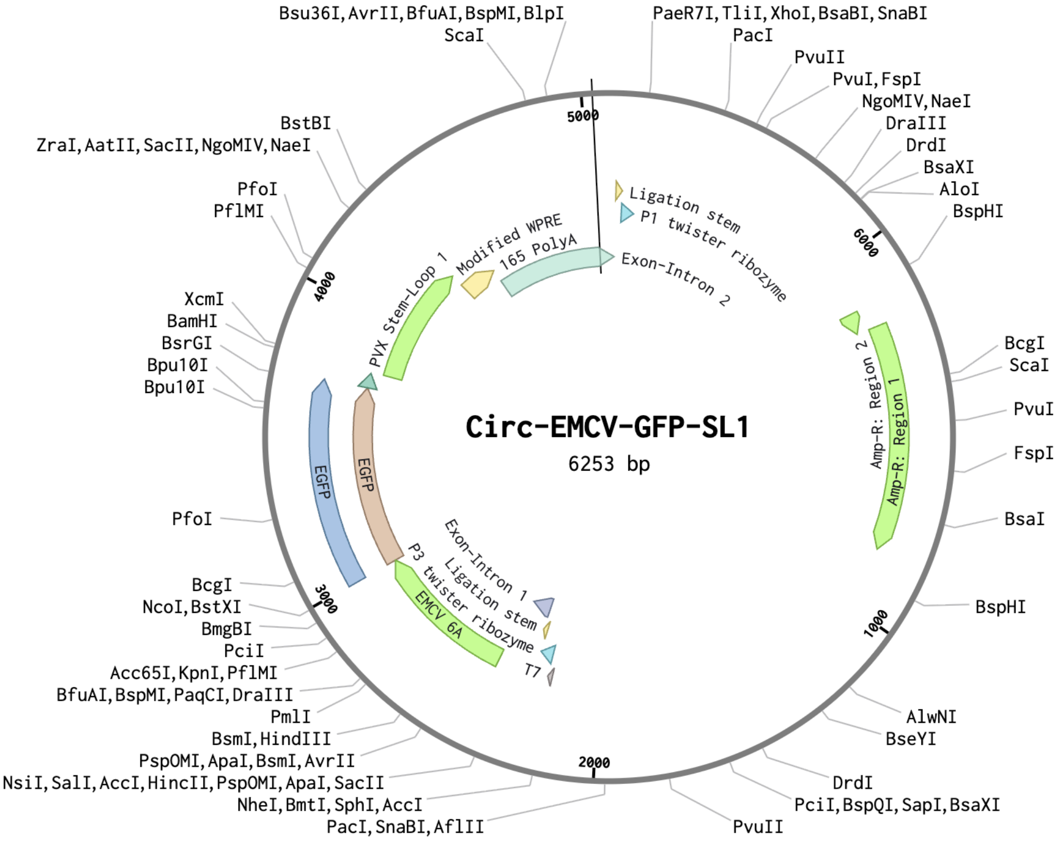


Sequence:

**AACGCTGGTGAAAGTAAAAGATGCTGAAGATCAGTTGGGTGCACGAGTGGGTTACATCGAACTGGATCTCAATAGTG
GTAAGATCCTTGAGAGTTTTCGCCCCGAAGAACGTTTTCCAATGATGAGCACTTTTAAAGTTCTGCTATGTGGCGCG
GTATTATCCCGTATTGACGCCGGGCAAGAGCAACTCGGTCGCCGCATACACTATTCTCAGAATGACTTGGTTGAGTA
CTCACCAGTCACAGAAAAGCATCTTACGGATGGCATGACAGTAAGAGAATTATGCAGTGCTGCCATAACCATGAGTG
ATAACACTGCGGCCAACTTACTTCTGACAACGATCGGAGGACCGAAGGAGCTAACCGCTTTTTTGCACAACATGGGG
GATCATGTAACTCGCCTTGATCGTTGGGAACCGGAGCTGAATGAAGCCATACCAAACGACGAGCGTGACACCACGAT
GCCTGTAGTAATGGTAACAACGTTGCGCAAACTATTAACTGGCGAACTACTTACTCTAGCTTCCCGGCAACAATTAA
TAGACTGGATGGAGGCGGATAAAGTTGCAGGACCACTTCTGCGCTCGGCCCTTCCGGCTGGCTGGTTTATTGCTGAT
AAATCTGGAGCCGGTGAGCGTGGGTCTCGCGGTATCATTGCAGCACTGGGGCCAGATGGTAAGCCCTCCCGTATCGT
AGTTATCTACACGACGGGGAGTCAGGCAACTATGGATGAACGAAATAGACAGATCGCTGAGATAGGTGCCTCACTGA
TTAAGCATTGGTAACTGTCAGACCAAGTTTACTCATATATACTTTAGATTGATTTAAAACTTCATTTTTAATTTAAA
AGGATCTAGGTGAAGATCCTTTTTGATAATCTCATGACCAAAATCCCTTAACGTGAGTTTTCGTTCCACTGAGCGTC
AGACCCCGTAGAAAAGATCAAAGGATCTTCTTGAGATCCTTTTTTTCTGCGCGTAATCTGCTGCTTGCAAACAAAAA
AACCACCGCTACCAGCGGTGGTTTGTTTGCCGGATCAAGAGCTACCAACTCTTTTTCCGAAGGTAACTGGCTTCAGC
AGAGCGCAGATACCAAATACTGTCCTTCTAGTGTAGCCGTAGTTAGGCCACCACTTCAAGAACTCTGTAGCACCGCC
TACATACCTCGCTCTGCTAATCCTGTTACCAGTGGCTGCTGCCAGTGGCGATAAGTCGTGTCTTACCGGGTTGGACT
CAAGACGATAGTTACCGGATAAGGCGCAGCGGTCGGGCTGAACGGGGGGTTCGTGCACACAGCCCAGCTTGGAGCGA
ACGACCTACACCGAACTGAGATACCTACAGCGTGAGCTATGAGAAAGCGCCACGCTTCCCGAAGGGAGAAAGGCGGA
CAGGTATCCGGTAAGCGGCAGGGTCGGAACAGGAGAGCGCACGAGGGAGCTTCCAGGGGGAAACGCCTGGTATCTTT
ATAGTCCTGTCGGGTTTCGCCACCTCTGACTTGAGCGTCGATTTTTGTGATGCTCGTCAGGGGGGCGGAGCCTATGG
AAAAACGCCAGCAACGCGGCCTTTTTACGGTTCCTGGCCTTTTGCTGCGGTTTTGCTCACATGTTCTTTCCTGCGTT
ATCCCCTGATTCTGTGGATAACCGTATTACCGCCTTTGAGTGAGCTGATACCGCTCGCCGCAGCCGAACGACCGAGC**

GCAGCGAGTCAGTGAGCGAGGAAGCGGAAGAGCGCCCAATACGCAAACCGCCTCTCCCCGCGCGTTGGCCGATTCAT
TAATGCAGCTGGCACGACAGGTTTCCCGACTGGAAAGCGGGCAGTGAGCGCAACGCAATTAATGTGAGTTAGCTCAC
TCATTAGGCACCCCAGGCTTTACACTTTATGCTTCCGGCTCGTATGTTGTGTGGAATTGTGAGCGGATAACAATTTC
ACACAGGAAACAGCTATGACCATGATTACGCCAGATTTAATTAAGGCTGCGCGCTCGCTCGCTCACTGAGGCCGCCC
GGGCAAAGCCCGGGCGTCGGGCGACCTTTGGTCGCCCGGCCTCAGTGAGCGAGCGAGCGCGCAGAGAGGGAGTGGCC
AACTCCATCACTAGGGGTTCCTTGTAGTTAATGATTAACCCGCCATGCTACTTATCTACGTAGCCATGCTCTAGGAA
GATCGGAATTCGCCCTTAAGCTAGCATGCTAATACGACTCACTATAGGGCCATCAGTCGCCGGTCCCAAGCCCGGAT
AAAATGGGAGGGGGCGGGAAACCGCCTAACCATGCCGACTGATGGCAGCTATTATCGAGCGAACGCCTTATGCGATG
AAAGTCGCACGTAGGGTGTAGACCAAGCGAAATCCTATGCATTTAGGATAGTGAGGTATAGCAAAGGAGAAGTCGAC
GGGCCCGCGGAATTCCGCCCCCCCCCCCTCTCCCTCCCCCCCCCCTAACGTTACTGGCCGAAGCCGCTTGGAATAAG
GCCGGTGTGCGTTTGTCTATATGTTATTTTCCACCATATTGCCGTCTTTTGGCAATGTGAGGGCCCGGAAACCTGGC
CCTGTCTTCTTGACGAGCATTCCTAGGGGTCTTTCCCCTCTCGCCAAAGGAATGCAAGGTCTGTTGAATGTCGTGAA
GGAAGCAGTTCCTCTGGAAGCTTCTTGAAGACAAACAACGTCTGTAGCGACCCTTTGCAGGCAGCGGAACCCCCCAC
CTGGCGACAGGTGCCTCTGCGGCCAAAAGCCACGTGTATAAGATACACCTGCAAAGGCGGCACAACCCCAGTGCCAC
GTTGTGAGTTGGATAGTTGTGGAAAGAGTCAAATGGCTCTCCTCAAGCGTATTCAACAAGGGGCTGAAGGATGCCCA
GAAGGTACCCCATTGTATGGGATCTGATCTGGGGCCTCGGTGCACATGCTTTACATGTGTTTAGTCGAGGTTAAAAA
ACGTCTAGGCCCCCCGAACCACGGGGACGTGGTTTTCCTTTGAAAAACACGATGATAATATGGCCACAACCATGGTG
AGCAAGGGCGAGGAGCTGTTCACCGGGGTGGTGCCCATCCTGGTCGAGCTGGACGGCGACGTAAACGGCCACAAGTT
CAGCGTGTCCGGCGAGGGCGAGGGCGATGCCACCTACGGCAAGCTGACCCTGAAGTTCATCTGCACCACCGGCAAGC
TGCCCGTGCCCTGGCCCACCCTCGTGACCACCCTGACCTACGGCGTGCAGTGCTTCAGCCGCTACCCCGACCACATG
AAGCAGCACGACTTCTTCAAGTCCGCCATGCCCGAAGGCTACGTCCAGGAGCGCACCATCTTCTTCAAGGACGACGG
CAACTACAAGACCCGCGCCGAGGTGAAGTTCGAGGGCGACACCCTGGTGAACCGCATCGAGCTGAAGGGCATCGACT
TCAAGGAGGACGGCAACATCCTGGGGCACAAGCTGGAGTACAACTACAACAGCCACAACGTCTATATCATGGCCGAC
AAGCAGAAGAACGGCATCAAGGTGAACTTCAAGATCCGCCACAACATCGAGGACGGCAGCGTGCAGCTCGCCGACCA
CTACCAGCAGAACACCCCCATCGGCGACGGCCCCGTGCTGCTGCCCGACAACCACTACCTGAGCACCCAGTCCGCCC
TGAGCAAAGACCCCAACGAGAAGCGCGATCACATGGTCCTGCTGGAGTTCGTGACCGCCGCCGGGATCACTCTCGGC
ATGGACGAGCTGTACAAGTAATTGTTACACACCCGCTTGAAAAAGCAAGTCTGACAAAAGGCCAAAGTGCGCGAGGG
CCACGGATCCAATCAACCTCTGGATTACAAAATTTGTGAAAGATTGACTGGTATTCTTAACTATGTTGCTCCTTTTA
CGCTATGTGGATACGCTGCTTTAATGCCTTTGTATCATGCTATTGCTTCCCGTATGGCTTTCATTTTCTCCTCCTTG
TATAAATCCTGGTTGCTGTCTCTTTATGAGGAGTTGTGGCCCGTTGTCAGGCAACGTGGCGTGGTGTGCACTGTGTT
TGCTGACGCAACCCCCACTGGTTGGGGCATTGCCACCACCTGTCAGCTCCTTTCCGGGACTTTCGCTTTCCCCCTCC
CTATTGCCACGGCGGAACTCATCGCCGCCTGCCTTGCCCGCTGCTGGACAGGGGCTCGGCTGTTGGGCACTGACAAT
TCCGTGGTGTTGTCGGGGAAATCATCGTCCTTTCCTTGGCTGCTCGCCTGTGTTGCCACCTGGATTCTGCGCGGGAC
GTCCTTCTGCTACAATCCAGCGGACCTTCCTTCCCGCGGCCTGCTGCCGGCTCTGCGGCCTCTTCCGCGTCTTCGAA
AAAAAAAAAAAAAAAAAAAAAAAAAAAAAAAAAAAAAAAAAAAAAAAAAAAAAAAAAAAAAAAAAAAAAAAAAAAAA
AAAAAAAAAAAAAAAAAAAAAAAAAAAAAAAAAAAAAAAAAAAAAAAAAAAAAAAAAAAAAAAAAAAAAAAAAAAAA
AAAAAAAAAAAACTATAGCCATACAATAAAAGTGCGAAACGTTATCCTATAAGTAAGAAAGTTTTAAAATTTTCTTA
CGAAAAGGATAGAACTTAAAAGTTCTAACTGTTCTACTAAAGTAATAAGTGAAAATCTTATTTAAAGCAAACAACCA
AGTAGCTTTAAGTCTAAGTCCCCTACACAAGTTTTATACTACTATGCAAAACTTGTGAAGCTAGGTAAGGTCGTAAT
CCGTGAAAGTCGGATGCGGGGCTCCTTAAAAGATTACTATGGTAAACATAAGCTAATCCATTAAGATGCGATTTATA
TGTATTTTATACTGTTAAATATTTTTGTGCTTGTGGCTTGGTATAAAACAGTTAAGATGAAGTACTTAACTGGTTTT
GGAATAATTGGTTGTTAAACTAAAACATTATAAATCGTTAGTGGATACCTAAGGTAATCAAAAATAGGGATAGGTAG
AATGGAACGTTTGATGCTGTATATGAAGAGGTTTAGTAGAACCTAGGACACATATACGGGCTCAGCAGGTTCATAGT
AGCTATGATACTCAGCCGGAAGTCAAATTAATTTTGAAATACTTCTATGGTAACATAGGAGAAGGATAAAACTGAGT
GAGCCAAGGAACCTAGTCGGTAATAGCTGCCATCAGTCGGCGTGGACTGTAGAACACTGCCAATGCCGGTCCCAAGC
CCGGATAAAAGTGGAGGGTACAGTCCACGCTTTTTTTCTCGAGTTAAGGGCGAATTCCCGATAAGGATCTTCCTAGA
GCATGGCTACGTAGATAAGTAGCATGGCGGGTTAATCATTAACTACAAGGAACCCCTAGTGATGGAGTTGGCCACTC
CCTCTCTGCGCGCTCGCTCGCTCACTGAGGCCGGGCGACCAAAGGTCGCCCGACGCCCGGGCTTTGCCCGGGCGGCC
TCAGTGAGCGAGCGAGCGCGCAGCCTTAATTAACCTAATTCACTGGCCGTCGTTTTACAACGTCGTGACTGGGAAAA
CCCTGGCGTTACCCAACTTAATCGCCTTGCAGCACATCCCCCTTTCGCCAGCTGGCGTAATAGCGAAGAGGCCCGCA
CCGATCGCCCTTCCCAACAGTTGCGCAGCCTGAATGGCGAATGGACGCGCCCTGTAGCGGCGCATTAAGCGCGGCG
GGTGTGGTGGTTACGCGCAGCGTGACCGCTACACTTGCCAGCGCCCTAGCGCCCGCTCCTTTCGCTTTCTTCCCTTC
CTTTCTCGCCACGTTCGCCGGCTTTCCCCGTCAAGCTCTAAATCGGGGGCTCCCTTTAGGGTTCCGATTTAGTGCTT
TACGGCACCTCGACCCCAAAAAACTTGATTAGGGTGATGGTTCACGTAGTGGGCCATCGCCCTGATAGACGGTTTTT
CGCCCTTTGACGTTGGAGTCCACGTTCTTTAATAGTGGACTCTTGTTCCAAACTGGAACAACACTCAACCCTATCTC
GGTCTATTCTTTTGATTTATAAGGGATTTTGCCGATTTCGGCCTATTGGTTAAAAAATGAGCTGATTTAACAAAAAT
TTAACGCGAATTTTAACAAAATATTAACGTTTATAATTTCAGGTGGCATCTTTCGGGGAAATGTGCGCGGAACCCCT

ATTTGTTTATTTTTCTAAATACATTCAAATATGTATCCGCTCATGAGACAATAACCCTGATAAATGCTTCAATAATA
TTGAAAAAGGAAGAGTATGAGTATTCAACATTTCCGTGTCGCCCTTATTCCCTTTTTTGCGGCATTTTGCCTTCCTG
TTTTTGCTCACCCAGA

## pCMV-T7-PVX-Genome

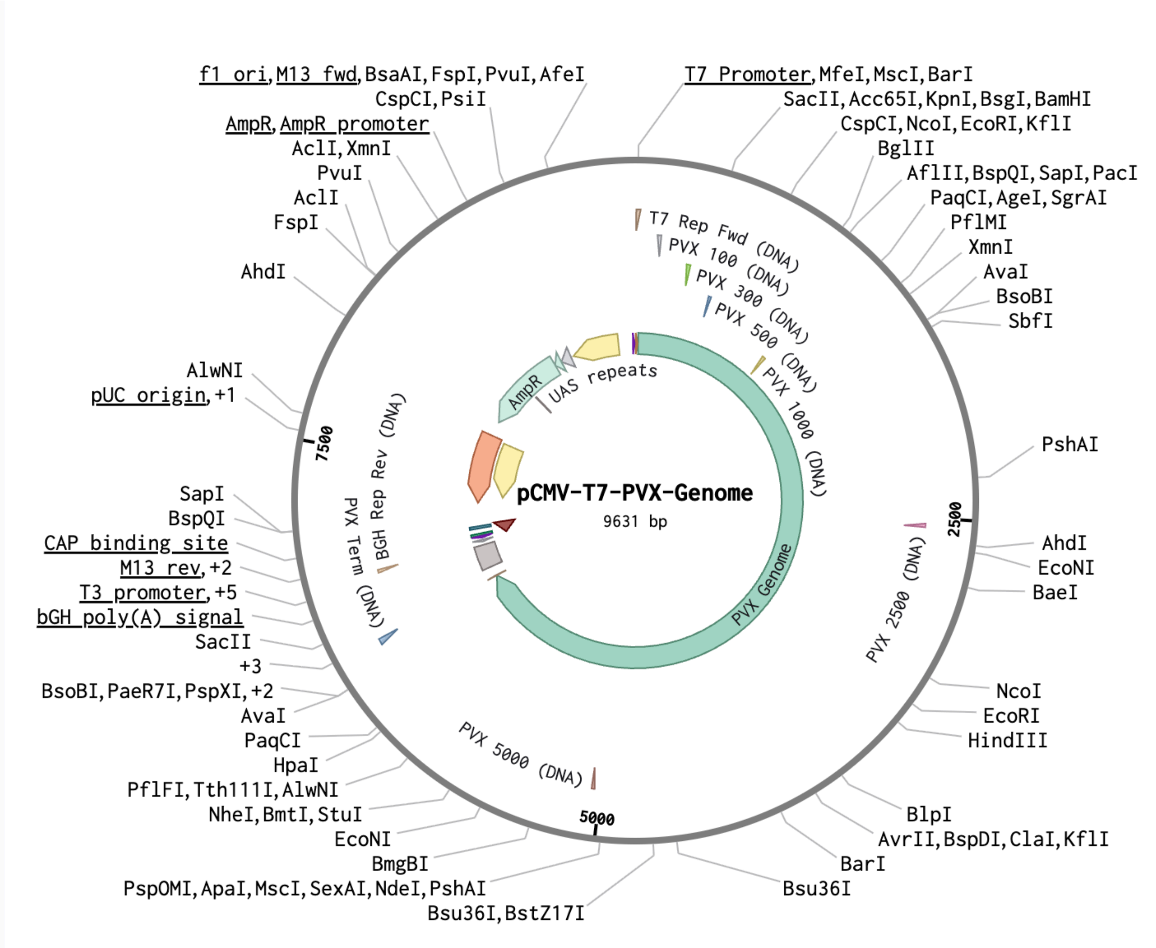


Sequence:

CTGCAGTAATACGACTCACTATAAGAGCCGAAAACTAAACCATACACCACCAACACAACCAAACCCACCACGCCCAA
TTGTTACACACCCGCTTGGAAAAGCAAGTCTAACAAATGGCCAAAGTGCGCGAGGTTTACCAATCCTTTACAGACTC
CACCACAAAAACTCTCATCCAAGATGAGGCTTATAGAAATATTCGCCCCATCATGGAAAAACACAAACTAGCTAACC
CTTACGCTCAAACGGTTGAAGCGGCTAATGATCTAGAGGGGTTCGGCATAGCCACCAATCCCTATAGCATTGAATTG
CATACACATGCAGCCGCTAAGACCATAGAGAATAAACTTCTAGAGGTGCTTGGTTCCATCCTACCACAAGAACCTGT
TACATTTATGTTCCTTAAACCCAGGAAGCTAAACTACATGAGAAGAAACCCGCGGATCAAGGACATTTTCCACAATG
TTGCCATTGAACCGAGAGACGTAGCAAGGTACCCCAAGGAAACAATAATTGACAAACTCACAGAGATCACAACGGAA
ACAGCATACATTAGTGATACTCTGCACTTCTTGGATCCGAGCTACATAGTGGAGACATTCCAAAACTGCCCAAAACT
GCAAACATTGTATGCGACCTTAGTTCTCCCCGTTGAGGCAGCCTTCAAAATGGAAAGCACTCACCCGAACATATACA
GCCTCAAATACTTCGGAGATGGTTTCCAGTATATACCAGGCAACCATGGTGGTGGGGCATACCATCATGAATTCGCT
CATTTACAATGGCTCAAAGTGGGAAAGATCAAGTGGAGGGACCCCAAGGATAGCTTTCTCGGACATCTCAATTACAC
GACTGAGCAGGTTGAGATGCACACAGTGACAGTACAGTTGCAGGAATCGTTTGCGGCAAACCACTTGTACTGCATTA
GGAGAGGAGACTTGCTCACACCGGAGGTGCGTACTTTCGGCCAACCTGACAGGTATGTGATTCCACCACAGATCTTT

CTCCCAAAAGTTCACAACTGCAAGAAGCCGATTCTTAAGAAAACTATGATGCAGCTCTTCTTGTATGTTAGGACAGT
CAAGGTCGCAAAAAATTGTGACATCTTTGCCAAAGTCAGACAATTAATTAAATCATCTGACTTAGACAAATATTCTG
CTGTGGAACTGGTTTACTTAGTAAGCTATATGGAGTTCCTTGCCGATTTACAAGCTACCACCTGCTTCTCAGACACA
CTCTCTGGTGGCTTGCTAACAAAGACCCTTGCACCGGTGAGGGCTTGGATACAAGAAAAGAAGATGCAGCTGTTTGG
TCTTGAGGACTACGCGAAGTTAGTCAAAGCAGTTGATTTCCACCCGGTGGATTTTTCTTTCAAAGTGGAAACTTGGG
ACTTCAGATTCCACCCCTTGCAAGCGTGGAAAGCCTTCCGACCAAGGGAAGTGTCGGATGTAGAGGAAATGGAAAGT
TTGTTCTCAGATGGGGACCTGCTTGATTGCTTCACAAGAATGCCAGCTTATGCAGTAAACGCAGAGGAAGATTTAGC
TACAATCAGGAAAACGCCCGAGATGGATGTCGGTCAAGAAGTTAAAGAGCCTGCAGGAGACAGAAATCAATACTCAA
ACCCTGCAGAAACTTTCCTCAACAAGCTCCACAGGAAACACAGTAGGGAGGTGAAACACCAGGCCGTAAAGAAAGCT
AAACGCCTAGCTGAAATCCAGGAGTCCATGAGAGCTGAAGGTGAGGCCGAACCAAATGAGACGAGCGGGGGCATGGG
GGCAATACCCAGCAACGCCGAACTTCCCGGCACGAGTGATGCCAGACAAGAACTCACACTCCCAACCACTAAACCTG
TTCCTGCAAGGTGGGAAGATGCTTCATTCACAGATTCTAGTGTGGAAGAGGAGCAGGTAAGACTCCTTGGAGAAGAA
GCAGTGAAAACAGCGACGCAGCAAGTCATCGAAGGACTCCCTTGGAAACACTGGATTCCTCAACTAAATGCTGTTGG
ATTCAAGGCGCTGTTAATCCAGAGGGATAGGAGTGGAACGATGATCATGCCCATCACAGAAATGGTCTCCGGGTTGG
AAAAAGAGGACTTCCCGGAAGGAACTCCAAAAGAGTTGGCACGAGAATTACTCGTTATGAACAGAAGCCCTGCCACC
ATCCCTTTGGACCTGCTTAGAGCCAGAGACTACGGCAGTGATGTAAAGAACAAGAGAATTGGTGCCATCACAAAGAC
ACAGGCAACGAGTTGGGGCGAGTACTTAACAGGAAAGATAGAAAGCCTGACTGAGAGGAAAGTTGCGACTTGTGTCA
TTCATGGAGCTGGAGGCTCTGGGAAAAGTCATGCCATCCAGAAGGCACTGAGAGAAATTGGCAAGGGCTCGGACATC
ACTGTAGTCCTGCCGACCAATGAACTGCGGCTAGATTGGAGTAAGAAGGTGCCTAACACTGAACCCTATATGTTCAA
GACCTACGAAAAGGCGTTAATTGGGGGAACAGGCAGCATAGTCATCTTTGACGATTACTCAAAACTTCCTCCCGGTT
ACATAGAAGCCTTAGTCTGTTTCTACTCCAAAATCAAGCTAATCATTCTAACAGGAGATAGCAGACAGAGCGTCTAC
CATGAAACTGCTGAGGACGCCTCCATCAGGCATTTGGGGCCAGCGACAGAGTACTTCTCAAAATACTGCCGATACTA
TCTCAATGCTACACACCGCAACAAGAAAGACCTTGCGAACATGCTTGGTGTCTACAGTGAGAGAACGGGAGTCACTG
AAATCAGCATGAGCGCCGAGTTTTTAGAAGGAATCCCAACTTTAGTACCCTCGGATGAGAAGAGAAAGCTGTACATG
GGCACCGGGAGGAATGACACATTCACATACGCTGGATGCCAGGGGCTAACTAAGCCGAAAGTACAAATAGTGTTGGA
CCACAACACCCAAGTGTGTAGTGCGAATGTGATGTACACGGCACTTTCTAGAGCCACCGATAGGATTCACTTCGTGA
ACACAAGTGCAAACTCCTCGGCCTTCTGGGAAAAGTTGGACAGCACCCCTTACCTCAAGACTTTCCTATCAGTGGTG
AGAGAACAAGCACTCAGGGAGTATGAGCCGGCAGAGGCAGAGCCAATTCAAGAGCCTGAGCCCCAGACACACATGTG
TGTCGAGAATGAGGAGTCCGTGCTAGAAGAGTACAAAGAGGAACTCTTGGAAAAGTTTGACAGAGAGATACACTCTG
AATCCCATGGTCATTCAAACTGTGTCCAAACTGAAGACACAACCATTCAGTTGTTTTCGCATCAACAAGCAAAAGAT
GAGACCCTCCTCTGGGCGACCATAGATGCGCGGCTCAAGACTAGCAATCAAGAGGCAAACTTCCGAGAATTCCTGAG
CAAGAAGGACATTGGGGACGTTCTGTTTTTAAACTACCAAAAAGCTATGGGTTTGCCCAAAGAGCGTATTCCTTTTT
CCCAAGAGGTCTGGGAAGCTTGTGCCCACGAAGTACAAAGCAAGTACCTCAGTAAGTCAAAGTGCAACTTGATCAAT
GGGACTGTGAGACAGAGCCCAGACTTCGATGAAAACAAGATTATGGTATTCCTCAAGTCGCAGTGGGTCACAAAGGT
GGAAAAACTAGGTCTACCCAAGATTAAGCCAGGTCAAACCATAGCAGCCTTTTACCAGCAGACTGTGATGCTTTTTG
GAACTATGGCTAGGTACATGCGATGGTTCAGACAGGCTTTCCAGCCAAAAGAAGTCTTCATAAACTGTGAGACTACG
CCAGAAGACATGTCTGTATGGGCCTTGAACAACTGGAATTTCAGCAGACCTAGCTTAGCTAATGACTACACAGCTTT
CGACCAGTCTCAGGATGGAGCTATGCTGCAATTTGAGGTGCTCAAAGCCAAGCACCACTGCATACCAGAGGAAATCA
TCCAAGCATACATAGACATTAAGACCAATGCACAGATTTTCCTAGGCACATTATCGATTATGCGCCTGACTGGTGAG
GGTCCCACTTTTGATGCAAACACTGAGTGCAACATAGCTTACACCCACACAAAGTTTGACATCCCAGCCGGAACTGC
TCAAGTTTATGCAGGAGACGACTCCGCACTGGATTGCGTTCCAGAAGTGAAGCATAGTTTCCACAGGCTTGAAGACA
AATTACTCCTCAAGTCAAAGCCTGTAATCACGCAGCAAAAGAAAGGCAGTTGGCCTGAGTTTTGTGGTTGGCTGATC
ACACCAAAAGGGGTAATGAAAGACCCAATTAAGCTCCATGTTAGCTTAAAATTGGCCGAAGCTAAGGGTGAACTCAA
GAAATGTCAAGATTCCTATGAAATTGATCTGAGTTATGCCTATGACCACAAGGACTCTCTGCATGACTTGTTCGATG
AGAAACAGTGTCAGGCACATACACTCACTTGCAGGACACTAATCAAGTCAGGGAGAGGCACTGTCTCACTTCCCCGC
CTCAGAAACTTTCTTTAACCGTTAATTTACCTTATAGATTTGAATAAGATGGATATTCTCATCAGTAGTTTGAAAAG
TTTAGGTTATTCTAGGACTTCTAAATCTTTAGATTCAGGACCTTTGGTAGTACATGCAGTAGCCGGAGCAGGTAAGT
CCACAGCCCTAAGGAAGTTGATCCTCAGACACCCAACATTCACCGTGCATACACTCGGTGTCCCTGACAAGGTGAGT
ATCAGAACTAGAGGCATACAGAAGCCAGGACCTATTCCTGAGGGCAATTTCGCAATCCTCGATGAGTATACTTTGGA
CAACACCACAAGGAACTCATACCAGGCACTTTTTGCTGACCCTTATCAGGCACCTGAGTTTAGCCTAGAGCCCCACT
TCTACTTGGAAACATCATTTCGAGTTCCGAGGAAAGTGGCAGATTTGATAGCTGGCTGTGGCTTCGATTTCGAGACT
AACTCACAGGAAGAAGGGCATTTAGAGATCACTGGCATATTCAAAGGGCCCCTACTTGGAAAGGTGATAGCCATTGA
TGAGGAGTCTGAGACAACACTGTCCAGGCATGGTGTTGAGTTTGTTAAGCCCTGCCAAGTGACTGGACTTGAGTTGA
AAGTAGTCACTATTGTGTCTGCCGCACCAATAGAGGAAATTGGCCAGTCCACAGCTTTCTACAACGCTATCACCAGG
TCAAAGGGATTGACATATGTCCGCGCAGGGACATAGACTGACCGCTCCGGTCAATTCTGAAAAAGTGTACATAGTAT
TAGGTCTATCATTTGCTTTAGTTTCAATTACTTTCCTGCTTTCTAGAAATAGTTTGCCCCACGTCGGTGACAACATT
CACAGCTTGCCACACGGAGGAGCTTACAGAGACGGCACCAAAGCAATCTTGTACAACTCCCCAAATCTAGGGTCACG

AGTGAGTCTACACAACGGAAAGAACGCAGCATTTGCTGCCGTTTTGCTACTGACTTTGCTGATCTATGGAAGTAAAT
ACATATCTCAACGCAATCATACTTGTGCTTGTGGTAACAATCATAGCAGTCATTAGTACTTCCTTAGTGAGGACTGA
ACCTTGTGTCATCAAGATTACTGGGGAATCAATCACAGTGTTGGCTTGCAAATTAGATGCAGAAACTATAAAAGCCA
TTGCCGATCTCAAGCCACTCTCCGTTGAACGGTTAAGTTTCCATTGATACTCGAAAGATGTCAGCACCAGCTAGCAC
AACACAGGCCACAGGGTCAACTACCTCAACTACCACGAAAACTGCAGGCGCAACTCCTGCCACAGCTTCAGGCCTGT
TCACCATCCCGGATGGGGATTTCTTTAGTACAGCTCGTGCCATAGTAGCCAGCAATGCTGTCGCAACAAATGAGGAC
CTCAGCAAGATTGAGGCTATTTGGAAGGACATGAAGGTGCCCACAGACACTATGGCACAGGCTGCTTGGGACTTAGT
CAGACACTGTGCTGATGTGGGATCATCTGCTCAAACAGAAATGATAGATACAGGTCCTTATTCCAACGGCATCAGCA
GAGCTAGACTGGCAGCAGCAATCAAAGAGGTGTGCACACTTAGGCAATTTTGCATGAAGTATGCTCCAGTGGTATGG
AACTGGATGTTAACTAACAACAGTCCACCTGCTAACTGGCAAGCACAAGGTTTCAAGCCTGAGCACAAATTCGCTGC
ATTCGACTTCTTCAATGGAGTCACCAACCCAGCTGCCATCATGCCCAAAGAGGGGCTCATCCGGCCACCGTCTGAAG
CTGAAATGAATGCTGCCCAAACTGCTGCTTTTGTGAAGATTACAAAGGCCAGGGCACAATCCAACGACTTTGCCAGC
CTAGATGCAGCTGTCACTCGAGGTCGTATCACTGGAACAACAACCGCTGAGGCTGTTGTCACTCTACCACCACCATA
ACTACGTCTACATAACCGACGCCTACCCCAGTTTCATAGTATTTTCTGGTTTGATTGTATGAATAATATAAATTCTA
GAGCTGGAGCCTCGGTAGCCGTTCCTCCTGCCCGCTGGGCCTCCCAACGGGCCCTCCTCCCCTCCTTGCACCGGCCC
TTCCTGGTCTTTGCCGCGGCTGTGCCTTCTAGTTGCCAGCCATCTGTTGTTTGCCCCTCCCCCGTGCCTTCCTTGAC
CCTGGAAGGTGCCACTCCCACTGTCCTTTCCTAATAAAATGAGGAAATTGCATCGCATTGTCTGAGTAGGTGTCATT
CTATTCTGGGGGGTGGGGTGGGGCAGGACAGCAAGGGGGAGGATTGGGAAGACAATAGCAGGCATGCTGGGGATGCG
GTGGGCTCTATGGTCGCCGGCGGTCGACGTACCCAGCTTTTGTTCCCTTTAGTGAGGGTTAATTGCGCGCTTGGCGT
AATCATGGTCATAGCTGTTTCCTGTGTGAAATTGTTATCCGCTCACAATTCCACACAACATACGAGCCGGAAGCATA
AAGTGTAAAGCCTGGGGTGCCTAATGAGTGAGCTAACTCACATTAATTGCGTTGCGCTCACTGCCCGCTTTCCAGTC
GGGAAACCTGTCGTGCCAGCTGCATTAATGAATCGGCCAACGCGCGGGGAGAGGCGGTTTGCGTATTGGGCGCTCTT
CCGCTTCCTCGCTCACTGACTCGCTGCGCTCGGTCGTTCGGCTGCGGCGAGCGGTATCAGCTCACTCAAAGGCGGTA
ATACGGTTATCCACAGAATCAGGGGATAACGCAGGAAAGAACATGTGAGCAAAAGGCCAGCAAAAGGCCAGGAACCG
TAAAAAGGCCGCGTTGCTGGCGTTTTTCCATAGGCTCCGCCCCCCTGACGAGCATCACAAAAATCGACGCTCAAGTC
AGAGGTGGCGAAACCCGACAGGACTATAAAGATACCAGGCGTTTCCCCCTGGAAGCTCCCTCGTGCGCTCTCCTGTT
CCGACCCTGCCGCTTACCGGATACCTGTCCGCCTTTCTCCCTTCGGGAAGCGTGGCGCTTTCTCATAGCTCACGCTG
TAGGTATCTCAGTTCGGTGTAGGTCGTTCGCTCCAAGCTGGGCTGTGTGCACGAACCCCCCGTTCAGCCCGACCGCT
GCGCCTTATCCGGTAACTATCGTCTTGAGTCCAACCCGGTAAGACACGACTTATCGCCACTGGCAGCAGCCACTGGT
AACAGGATTAGCAGAGCGAGGTATGTAGGCGGTGCTACAGAGTTCTTGAAGTGGTGGCCTAACTACGGCTACACTAG
AAGGACAGTATTTGGTATCTGCGCTCTGCTGAAGCCAGTTACCTTCGGAAAAAGAGTTGGTAGCTCTTGATCCGGCA
AACAAACCACCGCTGGTAGCGGTGGTTTTTTTGTTTGCAAGCAGCAGATTACGCGCAGAAAAAAAGGATCTCAAGAA
GATCCTTTGATCTTTTCTACGGGGTCTGACGCTCAGTGGAACGAAAACTCACGTTAAGGGATTTTGGTCATGAGATT
ATCAAAAAGGATCTTCACCTAGATCCTTTTAAATTAAAAATGAAGTTTTAAATCAATCTAAAGTATATATGAGTAAA
CTTGGTCTGACAGTTACCAATGCTTAATCAGTGAGGCACCTATCTCAGCGATCTGTCTATTTCGTTCATCCATAGTT
GCCTGACTCCCCGTCGTGTAGATAACTACGATACGGGAGGGCTTACCATCTGGCCCCAGTGCTGCAATGATACCGCG
AGACCCACGCTCACCGGCTCCAGATTTATCAGCAATAAACCAGCCAGCCGGAAGGGCCGAGCGCAGAAGTGGTCCTG
CAACTTTATCCGCCTCCATCCAGTCTATTAATTGTTGCCGGGAAGCTAGAGTAAGTAGTTCGCCAGTTAATAGTTTG
CGCAACGTTGTTGCCATTGCTACAGGCATCGTGGTGTCACGCTCGTCGTTTGGTATGGCTTCATTCAGCTCCGGTTC
CCAACGATCAAGGCGAGTTACATGATCCCCCATGTTGTGCAAAAAAGCGGTTAGCTCCTTCGGTCCTCCGATCGTTG
TCAGAAGTAAGTTGGCCGCAGTGTTATCACTCATGGTTATGGCAGCACTGCATAATTCTCTTACTGTCATGCCATCC
GTAAGATGCTTTTCTGTGACTGGTGAGTACTCAACCAAGTCATTCTGAGAATAGTGTATGCGGCGACCGAGTTGCTC
TTGCCCGGCGTCAATACGGGATAATACCGCGCCACATAGCAGAACTTTAAAAGTGCTCATCATTGGAAAACGTTCTT
CGGGGCGAAAACTCTCAAGGATCTTACCGCTGTTGAGATCCAGTTCGATGTAACCCACTCGTGCACCCAACTGATCT
TCAGCATCTTTTACTTTCACCAGCGTTTCTGGGTGAGCAAAAACAGGAAGGCAAAATGCCGCAAAAAAGGGAATAAG
GGCGACACGGAAATGTTGAATACTCATACTCTTCCTTTTTCAATATTATTGAAGCATTTATCAGGGTTATTGTCTCA
TGAGCGGATACATATTTGAATGTATTTAGAAAAATAAACAAATAGGGGTTCCGCGCACATTTCCCCGAAAAGTGCCA
CCTAAATTGTAAGCGTTAATATTTTGTTAAAATTCGCGTTAAATTTTTGTTAAATCAGCTCATTTTTTAACCAATAG
GCCGAAATCGGCAAAATCCCTTATAAATCAAAAGAATAGACCGAGATAGGGTTGAGTGTTGTTCCAGTTTGGAACAA
GAGTCCACTATTAAAGAACGTGGACTCCAACGTCAAAGGGCGAAAAACCGTCTATCAGGGCGATGGCCCACTACGTG
AACCATCACCCTAATCAAGTTTTTTGGGGTCGAGGTGCCGTAAAGCACTAAATCGGAACCCTAAAGGGAGCCCCCGA
TTTAGAGCTTGACGGGGAAAGCCGGCGAACGTGGCGAGAAAGGAAGGGAAGAAAGCGAAAGGAGCGGGCGCTAGGGC
GCTGGCAAGTGTAGCGGTCACGCTGCGCGTAACCACCACACCCGCCGCGCTTAATGCGCCGCTACAGGGCGCGTCCC
ATTCGCCATTCAGGCTGCGCAACTGTTGGGAAGGGCGATCGGTGCGGGCCTCTTCGCTATTACGCCAGCTGGCGAAA
GGGGGATGTGCTGCAAGGCGATTAAGTTGGGTAACGCCAGGGTTTTCCCAGTCACGACGTTGTAAAACGACGGCCAG
TGAGCG

## pHGWA-9xHis-PVX-CP

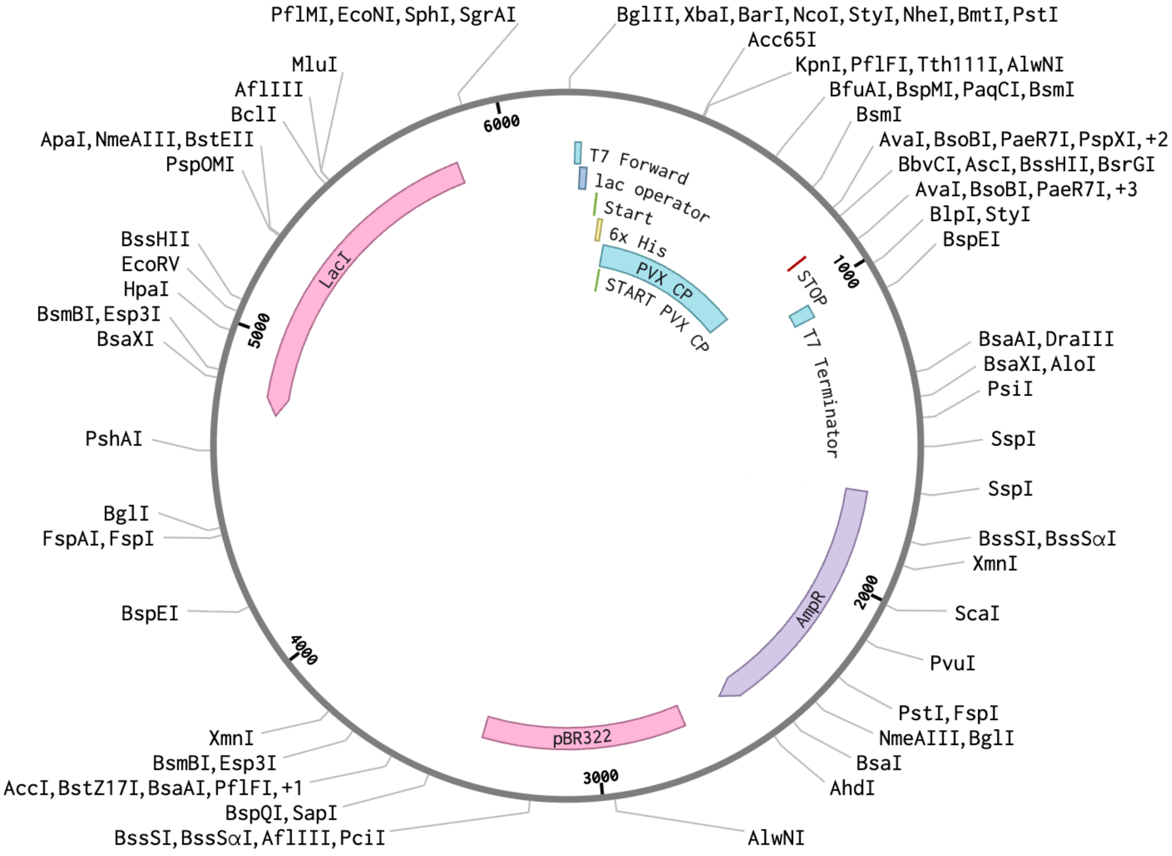


Sequence:

AGATCCAGATCTCGATCCCGCGAAATTAATACGACTCACTATAGGGGAATTGTGAGCGGATAACAATTCCCCTCTAG
AAATAATTTTGTTTAACTTTAAGAAGGAGATATACCATGGGCAGCAGCCATCATCATCATCATCATCATCATCACGG
TGAGAATCTTTATTTTCAGGGCATGTCAGCACCAGCTAGCACAACACAGACCATAGGGTCAACTACCTCAACTACCA
CAAAAACTGCAGGCGCAACTCCTGCCACAGCTTCAGGACTGTTCACCATCCCGGATGGGGATTTCTTTAACACAGCC
CGTGCCATAGTAGCCAGCAATGCCGTTGCAACGAATGAGGACCTCAGAAAGATTGAGGCTATTTGGAAAGACATGAA
GGTACCCACAGACACTATGGCACAGGCTGCTTGGGACTTGGTCAGACACTGTGCTGATGTGGGCTCATCTGCCCAAA
CAGAGATGATAGATACAGGTCCTTATTCCAATGGCATCAGCAGAGCTAGACTGGCAGCAGCGATTAAAGAGGTGTGC
ACACTTAGACAATTCTGCATGAAGTATGCCCCAGTGGTATGGAACTGGATGTTGACTAACAACAGTCCACCTGCTAA
CTGGCAAGCACAAGGTTTCAAGCCTGAGCACAAATTCGCTGCATTCGACTTCTTCAACGGAGTCACCAACCCAGCTG
CCATCATGCCCAAAGAGGGACTCATCCGGCCACCATCTGAAGCAGAAATGAATGCTGCCCAAACTGCTGCCTTTGTG
AAAATTACGAAGGCCAGGGCACAATCCAACGACTTTGCCAGCCTAGATGCGGCTGTCACTCGAGGTCGTATCACTGG
AACAACAACCGCTGAGGCTGTTGTCACTCTACCACCACCATAAAAGGGTGGGCGCGCCGACCCAGCTTTCTTGTACA
AAGTGGTGATGTACCTCGAGCACCACCACCACCACCACTGAGATCCGGCTGCTAACAAAGCCCGAAAGGAAGCTGAG
TTGGCTGCTGCCACCGCTGAGCAATAACTAGCATAACCCCTTGGGGCCTCTAAACGGGTCTTGAGGGGTTTTTTGCT
GAAAGGAGGAACTATATCCGGATTGGCGAATGGGACGCGCCCTGTAGCGGCGCATTAAGCGCGGCGGGTGTGGTGGT
TACGCGCAGCGTGACCGCTACACTTGCCAGCGCCCTAGCGCCCGCTCCTTTCGCTTTCTTCCCTTCCTTTCTCGCCA
CGTTCGCCGGCTTTCCCCGTCAAGCTCTAAATCGGGGGCTCCCTTTAGGGTTCCGATTTAGTGCTTTACGGCACCTC
GACCCCAAAAAACTTGATTAGGGTGATGGTTCACGTAGTGGGCCATCGCCCTGATAGACGGTTTTTCGCCCTTTGAC
GTTGGAGTCCACGTTCTTTAATAGTGGACTCTTGTTCCAAACTGGAACAACACTCAACCCTATCTCGGTCTATTCTT
TTGATTTATAAGGGATTTTGCCGATTTCGGCCTATTGGTTAAAAAATGAGCTGATTTAACAAAAATTTAACGCGAAT
TTTAACAAAATATTAACGTTTACAATTTCAGGTGGCACTTTTCGGGGAAATGTGCGCGGAACCCCTATTTGTTTATT

TTTCTAAATACATTCAAATATGTATCCGCTCATGAGACAATAACCCTGATAAATGCTTCAATAATATTGAAAAAGGA
AGAGTATGAGTATTCAACATTTCCGTGTCGCCCTTATTCCCTTTTTTGCGGCATTTTGCCTTCCTGTTTTTGCTCAC
CCAGAAACGCTGGTGAAAGTAAAAGATGCTGAAGATCAGTTGGGTGCACGAGTGGGTTACATCGAACTGGATCTCAA
CAGCGGTAAGATCCTTGAGAGTTTTCGCCCCGAAGAACGTTTTCCAATGATGAGCACTTTTAAAGTTCTGCTATGTG
GCGCGGTATTATCCCGTATTGACGCCGGGCAAGAGCAACTCGGTCGCCGCATACACTATTCTCAGAATGACTTGGTT
GAGTACTCACCAGTCACAGAAAAGCATCTTACGGATGGCATGACAGTAAGAGAATTATGCAGTGCTGCCATAACCAT
GAGTGATAACACTGCGGCCAACTTACTTCTGACAACGATCGGAGGACCGAAGGAGCTAACCGCTTTTTTGCACAACA
TGGGGGATCATGTAACTCGCCTTGATCGTTGGGAACCGGAGCTGAATGAAGCCATACCAAACGACGAGCGTGACACC
ACGATGCCTGCAGCAATGGCAACAACGTTGCGCAAACTATTAACTGGCGAACTACTTACTCTAGCTTCCCGGCAACA
ATTAATAGACTGGATGGAGGCGGATAAAGTTGCAGGACCACTTCTGCGCTCGGCCCTTCCGGCTGGCTGGTTTATTG
CTGATAAATCTGGAGCCGGTGAGCGTGGGTCTCGCGGTATCATTGCAGCACTGGGGCCAGATGGTAAGCCCTCCCGT
ATCGTAGTTATCTACACGACGGGGAGTCAGGCAACTATGGATGAACGAAATAGACAGATCGCTGAGATAGGTGCCTC
ACTGATTAAGCATTGGTAACTGTCAGACCAAGTTTACTCATATATACTTTAGATTGATTTAAAACTTCATTTTTAAT
TTAAAAGGATCTAGGTGAAGATCCTTTTTGATAATCTCATGACCAAAATCCCTTAACGTGAGTTTTCGTTCCACTGA
GCGTCAGACCCCGTAGAAAAGATCAAAGGATCTTCTTGAGATCCTTTTTTTCTGCGCGTAATCTGCTGCTTGCAAAC
AAAAAAACCACCGCTACCAGCGGTGGTTTGTTTGCCGGATCAAGAGCTACCAACTCTTTTTCCGAAGGTAACTGGCT
TCAGCAGAGCGCAGATACCAAATACTGTCCTTCTAGTGTAGCCGTAGTTAGGCCACCACTTCAAGAACTCTGTAGCA
CCGCCTACATACCTCGCTCTGCTAATCCTGTTACCAGTGGCTGCTGCCAGTGGCGATAAGTCGTGTCTTACCGGGTT
GGACTCAAGACGATAGTTACCGGATAAGGCGCAGCGGTCGGGCTGAACGGGGGGTTCGTGCACACAGCCCAGCTTGG
AGCGAACGACCTACACCGAACTGAGATACCTACAGCGTGAGCTATGAGAAAGCGCCACGCTTCCCGAAGGGAGAAAG
GCGGACAGGTATCCGGTAAGCGGCAGGGTCGGAACAGGAGAGCGCACGAGGGAGCTTCCAGGGGGAAACGCCTGGTA
TCTTTATAGTCCTGTCGGGTTTCGCCACCTCTGACTTGAGCGTCGATTTTTGTGATGCTCGTCAGGGGGGCGGAGCC
TATGGAAAAACGCCAGCAACGCGGCCTTTTTACGGTTCCTGGCCTTTTGCTGGCCTTTTGCTCACATGTTCTTTCCT
GCGTTATCCCCTGATTCTGTGGATAACCGTATTACCGCCTTTGAGTGAGCTGATACCGCTCGCCGCAGCCGAACGAC
CGAGCGCAGCGAGTCAGTGAGCGAGGAAGCGGAAGAGCGCCTGATGCGGTATTTTCTCCTTACGCATCTGTGCGGTA
TTTCACACCGCATATATGGTGCACTCTCAGTACAATCTGCTCTGATGCCGCATAGTTAAGCCAGTATACACTCCGCT
ATCGCTACGTGACTGGGTCATGGCTGCGCCCCGACACCCGCCAACACCCGCTGACGCGCCCTGACGGGCTTGTCTGC
TCCCGGCATCCGCTTACAGACAAGCTGTGACCGTCTCCGGGAGCTGCATGTGTCAGAGGTTTTCACCGTCATCACCG
AAACGCGCGAGGCAGCTGCGGTAAAGCTCATCAGCGTGGTCGTGAAGCGATTCACAGATGTCTGCCTGTTCATCCGC
GTCCAGCTCGTTGAGTTTCTCCAGAAGCGTTAATGTCTGGCTTCTGATAAAGCGGGCCATGTTAAGGGCGGTTTTTT
CCTGTTTGGTCACTGATGCCTCCGTGTAAGGGGGATTTCTGTTCATGGGGGTAATGATACCGATGAAACGAGAGAGG
ATGCTCACGATACGGGTTACTGATGATGAACATGCCCGGTTACTGGAACGTTGTGAGGGTAAACAACTGGCGGTATG
GATGCGGCGGGACCAGAGAAAAATCACTCAGGGTCAATGCCAGCGCTTCGTTAATACAGATGTAGGTGTTCCACAGG
GTAGCCAGCAGCATCCTGCGATGCAGATCCGGAACATAATGGTGCAGGGCGCTGACTTCCGCGTTTCCAGACTTTAC
GAAACACGGAAACCGAAGACCATTCATGTTGTTGCTCAGGTCGCAGACGTTTTGCAGCAGCAGTCGCTTCACGTTCG
CTCGCGTATCGGTGATTCATTCTGCTAACCAGTAAGGCAACCCCGCCAGCCTAGCCGGGTCCTCAACGACAGGAGCA
CGATCATGCGCACCCGTGGGGCCGCCATGCCGGCGATAATGGCCTGCTTCTCGCCGAAACGTTTGGTGGCGGGACCA
GTGACGAAGGCTTGAGCGAGGGCGTGCAAGATTCCGAATACCGCAAGCGACAGGCCGATCATCGTCGCGCTCCAGCG
AAAGCGGTCCTCGCCGAAAATGACCCAGAGCGCTGCCGGCACCTGTCCTACGAGTTGCATGATAAAGAAGACAGTCA
TAAGTGCGGCGACGATAGTCATGCCCCGCGCCCACCGGAAGGAGCTGACTGGGTTGAAGGCTCTCAAGGGCATCGGT
CGAGATCCCGGTGCCTAATGAGTGAGCTAACTTACATTAATTGCGTTGCGCTCACTGCCCGCTTTCCAGTCGGGAAA
CCTGTCGTGCCAGCTGCATTAATGAATCGGCCAACGCGCGGGGAGAGGCGGTTTGCGTATTGGGCGCCAGGGTGGTT
TTTCTTTTCACCAGTGAGACGGGCAACAGCTGATTGCCCTTCACCGCCTGGCCCTGAGAGAGTTGCAGCAAGCGGTC
CACGCTGGTTTGCCCCAGCAGGCGAAAATCCTGTTTGATGGTGGTTAACGGCGGGATATAACATGAGCTGTCTTCGG
TATCGTCGTATCCCACTACCGAGATATCCGCACCAACGCGCAGCCCGGACTCGGTAATGGCGCGCATTGCGCCCAGC
GCCATCTGATCGTTGGCAACCAGCATCGCAGTGGGAACGATGCCCTCATTCAGCATTTGCATGGTTTGTTGAAAACC
GGACATGGCACTCCAGTCGCCTTCCCGTTCCGCTATCGGCTGAATTTGATTGCGAGTGAGATATTTATGCCAGCCAG
CCAGACGCAGACGCGCCGAGACAGAACTTAATGGGCCCGCTAACAGCGCGATTTGCTGGTGACCCAATGCGACCAGA
TGCTCCACGCCCAGTCGCGTACCGTCTTCATGGGAGAAAATAATACTGTTGATGGGTGTCTGGTCAGAGACATCAAG
AAATAACGCCGGAACATTAGTGCAGGCAGCTTCCACAGCAATGGCATCCTGGTCATCCAGCGGATAGTTAATGATCA
GCCCACTGACGCGTTGCGCGAGAAGATTGTGCACCGCCGCTTTACAGGCTTCGACGCCGCTTCGTTCTACCATCGAC
ACCACCACGCTGGCACCCAGTTGATCGGCGCGAGATTTAATCGCCGCGACAATTTGCGACGGCGCGTGCAGGGCCAG
ACTGGAGGTGGCAACGCCAATCAGCAACGACTGTTTGCCCGCCAGTTGTTGTGCCACGCGGTTGGGAATGTAATTCA
GCTCCGCCATCGCCGCTTCCACTTTTTCCCGCGTTTTCGCAGAAACGTGGCTGGCCTGGTTCACCACGCGGGAAACG
GTCTGATAAGAGACACCGGCATACTCTGCGACATCGTATAACGTTACTGGTTTCACATTCACCACCCTGAATTGACT
CTCTTCCGGGCGCTATCATGCCATACCGCGAAAGGTTTTGCGCCATTCGATGGTGTCCGGGATCTCGACGCTCTCCC
TTATGCGACTCCTGCATTAGGAAGCAGCCCAGTAGTAGGTTGAGGCCGTTGAGCACCGCCGCCGCAAGGAATGGTGC

**ATGCAAGGAGATGGCGCCCAACAGTCCCCCGGCCACGGGGCCTGCCACCATACCCACGCCGAAACAAGCGCTCATGA
GCCCGAAGTGGCGAGCCCGATCTTCCCCATCGGTGATGTCGGCGATATAGGCGCCAGCAACCGCACCTGTGGCGCCG
GTGATGCCGGCCACGATGCGTCCGGCGTAGAGGATCG**
